# Morpholine-Substituted Tetrahydroquinoline Derivatives as Potential mTOR Inhibitors: Synthesis, Computational Insights, and Cellular Analysis

**DOI:** 10.3390/cancers17050759

**Published:** 2025-02-23

**Authors:** Rajdeep Dey, Suman Shaw, Ruchi Yadav, Bhumika D. Patel, Hardik G. Bhatt, Gopal Natesan, Abhishek B. Jha, Udit Chaube

**Affiliations:** 1 Department of Pharmaceutical Chemistry, Institute of Pharmacy, Nirma University, Ahmedabad 382481, Gujarat, India; 22ftphdp88@nirmauni.ac.in (R.D.); 20ftphdp70@nirmauni.ac.in (S.S.); bhumika.patel@nirmauni.ac.in (B.D.P.); hardikbhatt23@nirmauni.ac.in (H.G.B.); gopal@nirmauni.ac.in (G.N.); 2 Department of Pharmacology, Institute of Pharmacy, Nirma University, Ahmedabad 382481, Gujarat, India; 20ftphdp62@nirmauni.ac.in; 3 Department of Internal Medicine, Roy J.and Lucille A. Carver College of Medicine, University of IOWA, Iowa City, IA 52242, USA

**Keywords:** mTOR, lung cancer, tetrahydroquinolines, molecular dynamics simulation

## Abstract

**Backgrounds:** This study explores the design of substituted tetrahydroquinoline (THQ) derivatives and their synthesis as possible inhibitors of mTOR inhibitors for targeted cancer therapy. **Methods:** Inspired by the structural characteristics of known mTOR inhibitors, eight novel derivatives were synthesized, characterized using mass spectroscopy, ^1^H, and ^13^C NMR, and evaluated for anticancer activity. **Results:** Computational studies, including molecular docking and molecular dynamics (MD) simulations, highlighted the derivative’s strong binding interaction and stability within the mTOR active site. Assays for in vitro cytotoxicity showed strong and specific anticancer action against cell lines of triple-negative breast cancer, lung cancer, and breast cancer while causing negligible impact on healthy cells. **Conclusions:** Compound **10e** emerged as the most promising candidate, displaying exceptional activity against A549 cells (IC_50_ = 0.033 µM) and inducing apoptosis in a dose-dependent manner, surpassing standard agents, like Everolimus and 5-flurouracil. Structure–activity relationship analysis revealed that incorporating trifluoromethyl and morpholine moieties significantly enhanced selectivity and potency. MD simulations further validated these findings, confirming stable protein-ligand interactions and favorable dynamics over a 100-ns simulation period. Collectively, this study underscores the therapeutic potential of THQ derivatives, particularly compound **10e**, as promising mTOR inhibitors with potential applications in lung cancer treatment.

## Simple Summary

Cancer is one of the leading causes of death worldwide, and scientists are constantly searching for better treatments. This study focuses on developing new drug-like molecules that can block a key protein called mTOR, which plays a crucial role in cancer cell growth and survival. In this manuscript, we designed and synthesized eight new tetrahydroquinoline analogs and evaluated their ability to stop the growth of lung and breast cancer cells while ensuring that they do not harm normal cells. The most promising compound, **10e**, showed exceptional cytotoxicity for lung cancer cells at very low doses and worked even better than existing cancer drugs, like Everolimus and 5-fluorouracil. Advanced computer simulations helped confirm how these compounds attach to the mTOR protein and block its function. Further investigation revealed that these compounds trigger a natural process called apoptosis, which results in programmed cell death. These findings suggest that the novel THQ derivatives, especially **10e**, could be strong candidates for future cancer treatments. With further testing, they may help create safer and more effective drugs for patients with lung and breast cancer.

## 1. Introduction

Cancer continues to be a major global health concern, with lung cancer being a leading cause of cancer-related fatalities [1,2]. The mTOR pathway plays a vital role in regulating cell growth, metabolism, and survival and is closely associated with cancer progression [3,4]. Targeting mTOR is a key focus in developing new cancer therapies because its dysregulation is common in lung cancer, leading to tumor growth, resistance to cell death, and spread of cancer [5]. mTOR is a serine/threonine kinase that operates in two distinct complexes, i.e., mTORC1, and mTORC2, responsible for controlling protein synthesis, autophagy, and cellular metabolism [6,7].

The search for strong and specific mTOR inhibitors has accelerated in recent years [8,9,10]. Despite their therapeutic utility, existing mTOR inhibitors, such as rapamycin and its analogs (rapalogs), are typically limited by their inability to effectively inhibit mTORC_1_ and their lack of efficacy against mTORC_2_ [11,12]. Furthermore, new resistance mechanisms emphasize the necessity of creative small compounds that can successfully target mTOR and bypass these restrictions. The potential for improved efficacy, selectivity, and bioavailability makes small molecule inhibitors with particular structural and pharmacophoric characteristics appealing options for cancer treatment [13].

In medicinal chemistry, tetrahydroquinoline derivatives are a flexible scaffold that exhibits a range of biological actions, such as antibacterial [14], anti-inflammatory [15], and anticancer effects [16]. It has been demonstrated that adding functional groups, like morpholine, improves the pharmacokinetic and pharmacodynamic characteristics of potential medications [17]. Morpholine’s ability to improve solubility, membrane permeability, and target interactions makes it a valuable pharmacophore in the design of novel therapeutic agents [18,19]. Understanding the therapeutic potential of these structural patterns, we set out to synthesize several tetrahydroquinoline derivatives that were replaced with morpholine as possible mTOR inhibitors.

The design of the novel THQ derivatives was guided by the structural and functional properties of mTOR, along with previous research on its inhibitors [20,21,22,23]. The binding score, stability, and interactions of the proposed derivatives with the mTOR active site (PDB ID: 4JT6) were predicted using in silico methods, such as molecular docking and molecular dynamics (MD) simulations [24]. Computational analysis strongly supported the synthesis of these compounds, which were later characterized through ^1^H NMR, ^13^C NMR, and mass spectrometry to verify their chemical structures, while HPLC was used to assess their purity.

## 2. Methodologies

### 2.1. Designing Rationale of Morpholine Substituted THQ Derivatives

The design strategy for morpholine-substituted tetrahydroquinoline (THQ) derivatives as potential mTOR inhibitors focused on optimizing key molecular features to enhance active site interactions. In the core scaffold, various fluorine/trifluoromethyl-substituted aromatic rings were incorporated, which is favorable for activity [20], with a morpholine-like substitution positioned at a distance of 1–2 carbon atoms from the THQ core to promote binding [19]. An amide linkage connects the aromatic ring, enabling hydrogen bond interactions with the TYR2225 residue, while the oxygen atom (X = O) facilitates additional hydrogen bond interactions with VAL2240 [25]. These structural modifications were strategically chosen to improve receptor–ligand interaction and specificity toward the mTOR active site.

**Figure 1:**
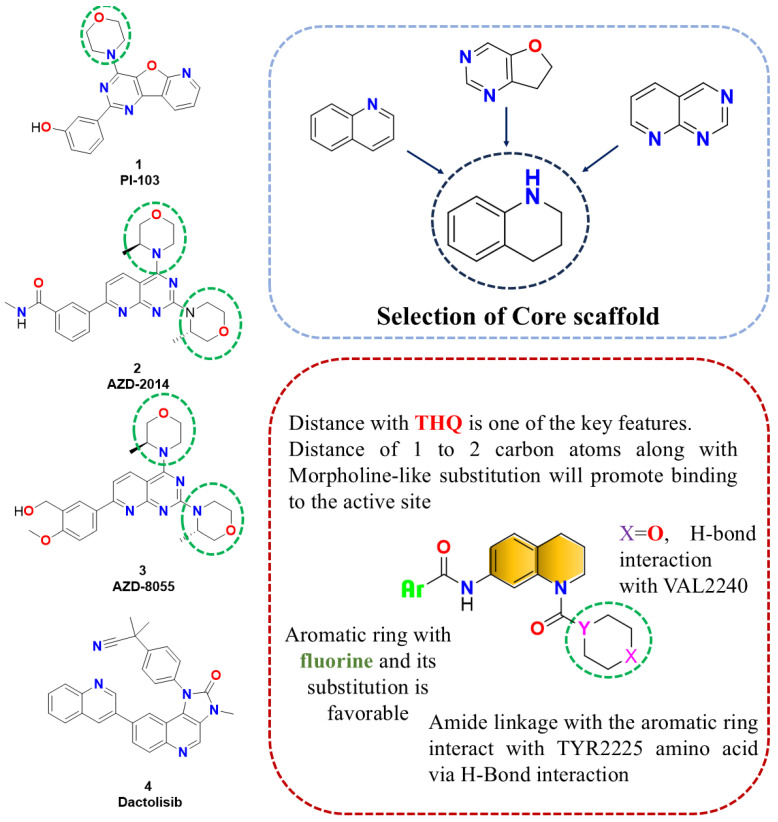
Strategic design of tetrahydroquinoline analogs, green circle represents morpholine-like substitutions that are crucial for mTOR activity.

Tetrahydroquinoline was chosen as the core scaffold based on bioisosteric principles and its structural characteristics with significant mTOR inhibitors, such as PI-103 (**1**), AZD-2014 (**2**), AZD-8055 (**3**), and dactolisib (**4**) [26,27,28,29]. PI-103 (**1**) features a dihydro benzofuran moiety, AZD2014 (**2**), and AZD8055 (**3**) incorporate quinazoline cores, respectively, while dactolisib (**4**) contains a fused quinoline structure. These scaffolds contribute to critical hydrogen bond interactions and π-stacking with residues in the mTOR active site [23]. However, quinoline, quinazoline, and 2,3-dihydrobenzofuran scaffolds were systematically replaced with tetrahydroquinoline due to their ability to maintain similar electronic and spatial properties while improving metabolic stability and reducing off-target effects [30,31]. Furthermore, the inclusion of a morpholine ring, which is typical in most clinical and marketed mTOR inhibitors, was critical for its role in increasing water solubility and facilitating specific interactions with mTOR’s ATP-binding pocket [32]. These structural modifications align with established pharmacophoric features, enabling potent and selective mTOR inhibition. Building on our earlier research that established a significant relationship between mTOR and tetrahydroquinoline [19,20,21,33,34], we designed a new set of derivatives to further explore and optimize this interaction. Specifically, a series of substituted N-(1-(morpholine-4-carbonyl) derivatives were synthesized, incorporating the tetrahydroquinoline scaffold for its established mTOR-binding potential, as illustrated in Figure 1.

### 2.2. Synthesis and Spectral Characterization of Designed THQ Derivatives

**Figure 2:**
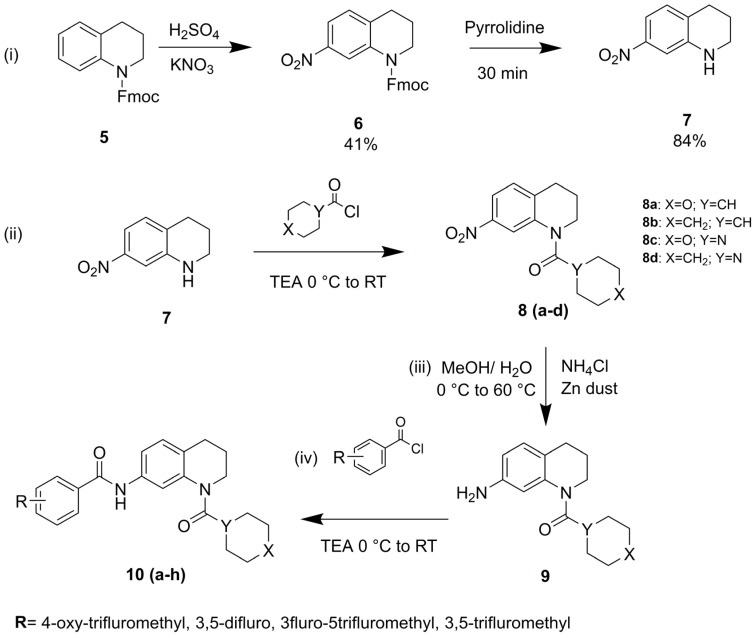
Synthetic scheme and procedure: reagents and Conditions: (**i**) nitration: KNO_3_/H_2_SO_4_ in DCM, stirred at 0 °C to room temperature (RT) for 2 h, followed by deprotection with pyrrolidine at RT. (**ii**) Amidation/coupling: triethylamine in DCM, stirred for 24 h. (**iii**) Reduction: Zn/NH_4_Cl in methanol, heated at 60 °C for 6 h. (**iv**) Final step: triethylamine in DCM, stirred for 1–2 h.

A well-defined synthetic scheme was developed to synthesize the designed THQ derivatives, as illustrated in Figure 2. The synthesis of intermediates and final compounds was carried out using borosilicate glassware and dry solvents. Specific steps in the procedure required heating and stirring, which were performed using a DIPALI Rota-mantle. Solvent removal was achieved with a BUCHI rotary vacuum evaporator, and the synthesized products were dried under an IR lamp for an appropriate duration. Once dried, all compounds were subjected to melting point determination using a VEEGO melting point apparatus. All tetrahydroquinoline (THQ) derivatives and intermediates were purified using column chromatography. The purity of the final compounds was assessed using high-performance liquid chromatography (HPLC, Agilent), confirming that all compounds used for in vitro screening exhibited a purity of over 95%. Structural characterization was performed using mass spectrometry (Agilent) and NMR spectroscopy, including ^1^H and ^13^C NMR (Bruker).

#### 2.2.1. Synthesis of 7-Nitro-1,2,3,4-tetrahydroquinoline

The nitrating mixture was prepared by dissolving KNO_3_ (1000 mmol, 0.142 g) in H_2_SO_4_ (1000 mmol, 0.074 mL) and stirring at 0 °C for 10–15 min. Dichloromethane was then added to the reaction mixture at the same temperature and stirred for an additional 15 min. While maintaining the temperature at 0 °C, Fmoc-protected THQ (5) (1000 mmol, 0.5 g), pre-dissolved in dichloromethane, was added dropwise. The reaction mixture was then stirred at room temperature for 2 h and 30 min. After completion, the reaction was quenched by pouring it over crushed ice, followed by extraction with dichloromethane and washing with brine. The product was obtained as a bright yellow solid (yield: 41%; mp: 146–148 °C). To remove the Fmoc protecting group, the crude product was treated with pyrrolidine at room temperature. After 30 min, the reaction mixture was extracted with dichloromethane and washed multiple times with water and brine [35,36].

6-nitro-tetrahydroquinoline and 7-nitro-tetrahydroquinoline (**7**) were formed with yields of 41% and 25%, respectively. The reaction products were analyzed by HPLC, where the retention times (RT) were 9.33 min for 7 nitro-THQ and 9.82 min for 6 nitro-THQ, indicating close elution profiles. The chromatogram is attached as Supplementary Figure S1. The regioisomers were successfully separated using preparative HPLC (instrument name: DSP Lab pure series Prep HPLC; DSP Instrument Pvt. Ltd., Hydrabad Telengana, India; column: C18, 5 μm, 20 × 250 Id.) [37,38].

#### 2.2.2. Synthesis of (7-Nitro-3,4-dihydroquinolin-1(2H)-yl)(tetrahydro-2H-pyran-4-yl)methanone (**8a**)

Compound 7 (1000 mmol, 0.2 g) was dissolved in dichloromethane, and trimethylamine (1000 mmol, 0.16 mL) was added dropwise under continuous stirring. After 30 min, tetrahydro-2H-pyran-4-carbonyl chloride (1000 mmol, 0.14 mL) was introduced into the reaction mixture. The reaction was stirred at room temperature for 24 h and then quenched by pouring it over crushed ice. The product was extracted using dichloromethane and subsequently washed with a bicarbonate solution, brine, and sodium sulfate. A light-yellow powder was obtained with a 66% yield; mp: 177–179 °C.

#### 2.2.3. Synthesis of Cyclohexyl(7-nitro-3,4-dihydroquinolin-1(2H)-yl)methanone (**8b**)

The procedure is the same as for **8a** only instead of tetrahydro-2H-pyran-4-carbonyl chloride, cyclohexanecarbonyl chloride was used in the reaction. 78% yield, mp: 161–163 °C.

#### 2.2.4. Synthesis of Morpholino(7-nitro-3,4-dihydroquinolin-1(2H)-yl)methanone (**8c**)

The procedure is the same as for **8a**, only instead of tetrahydro-2H-pyran-4-carbonyl chloride, morpholine-4-carbonyl chloride was used in the reaction. 39% yield, mp: 188–190 °C.

#### 2.2.5. Synthesis of (7-Nitro-3,4-dihydroquinolin-1(2H)-yl)(piperidin-1-yl)methanone (**8d**)

The procedure is the same as **8a**, only instead of tetrahydro-2H-pyran-4-carbonyl chloride, piperidine-1-carbonyl chloride was used in the reaction. 46% yield, mp: 175–176 °C.

#### 2.2.6. Synthesis of (7-Amino-3,4-dihydroquinolin-1(2H)-yl)(tetrahydro-2H-pyran-4-yl)methanone (**9a**)

A solution of ammonium chloride (7000 mmol, 0.5 g) was prepared in a 3:7 water-to-methanol ratio. Simultaneously, compound **8a** (1000 mmol, 0.3 g) was dissolved in methanol, followed by the addition of zinc dust (10,000 mmol, 0.43 g). The prepared ammonium chloride solution was then added to the reaction mixture. The nitro group was reduced to an amine by stirring the mixture at 60 °C for six hours. After the reaction was complete, methanol was removed, and the residue was dissolved in ethyl acetate. The product was extracted using a bicarbonate solution, dried over sodium sulfate, and purified by column chromatography.

Yield: 68%; mp: 196–198 °C.

#### 2.2.7. Synthesis of (7-Amino-3,4-dihydroquinolin-1(2H)-yl)(cyclohexyl)methanone (**9b**)

The procedure of synthesis of compound **9b** remained the same as that of the synthesis of compound **9a**; the only difference was that compound **8b** was used instead of **8a**.

Yield: 73%; mp: 200–202 °C.

#### 2.2.8. Synthesis of Morpholino(7-amino-3,4-dihydroquinolin-1(2H)-yl)methanone (**9c**)

The procedure for synthesizing compound **9c** remained the same as that for synthesizing compound **9a**; the only difference was that compound **8c** was used instead of **8a**.

Yield: 81%; mp: 160–162 °C.

#### 2.2.9. Synthesis of (7-Amino-3,4-dihydroquinolin-1(2H)-yl)(piperidin-1-yl)methanone (**9d**)

The procedure for synthesizing compound **9d** remained the same as that for synthesizing compound **9a**; the only difference was that compound **8d** was used instead of **8a**.

Yield: 77%; mp: 177–178 °C.

#### 2.2.10. Synthesis of N-(1-(Tetrahydro-2H-pyran-4-carbonyl)-1,2,3,4-tetrahydroquinolin-7-yl)-4-(trifluoromethoxy)benzamide (**10a**)

Compound **9a** (1.7 mmol, 0.5 g) was dissolved in dichloromethane (DCM), and trimethylamine (4.9 mmol, 0.46 mL) was added dropwise under continuous stirring. The mixture was stirred at room temperature for 30 min, followed by the addition of 4-(trifluoromethoxy)benzoyl chloride (1.6 mmol, 0.3 g). Stirring continued for an additional hour. After completion, the reaction mixture was poured over crushed ice, extracted with DCM, and dried over sodium sulfate, yielding a yellow-colored compound. The final product was purified by column chromatography.

Light yellow solid yield: 45.54%; mp: 217–219 °C; ^1^H NMR (400 MHz, CDCl_3_) δ PPM: 1.30–1.20 (m, 4H, -CH_2_), 1.57–1.51 (m, 2H, -CH_2_); 1.81–1.79 (m, 4H, -CH_2_), 1.96–1.91 (p, 2H, -CH_2_), 2.65–2.61 (t, 2H, -CH_2_); 3.74–3.71 (t, 2H, -CH_2_) 7.07 (s, 1H, Ar-CH); 7.66 (s, 2H, Ar-CH), 8.01 (s, 1H, Ar-CH), 8.35 (s, 2H, Ar-CH), 8.48 (s, 1H, Ar-CH), 9.03 (s, 1H, NH-CO); 13C NMR (100 MHz, CDCl_3_) δ PPM: 24.19, 26.26, 29.38, 29.67, 38.64, 67.11, 116.87, 117.23, 119.00, 120.74, 124.14, 128.94, 129.02, 133.24, 135.70, 151.68, 164.45, 174.78; MS (EI) *m*/*z* calculated for C_23_H_23_F_3_N_2_O_4_ 448.44; found 449.47 (M + 1); HPLC purity: 96.13%.

#### 2.2.11. Synthesis of N-(1-(Cyclohexanecarbonyl)-1,2,3,4-tetrahydroquinolin-7-yl)-4-(trifluoromethoxy)benzamide (**10b**)

The reaction process is identical to compound **10a**; however, **9b** was used to synthesize compound **10b** rather than **9a**.

Pale yellow solid yield: 61.45%; mp: 210–212 °C; ^1^H NMR (400 MHz, CDCl_3_) δ PPM: 1.35–1.27 (m, 4H, -CH_2_), 1.68–1.57 (m, 2H, -CH_2_); 1.83–1.80 (d, 4H, -CH_2_), 2.1–1.98 (p, 2H, -CH_2_), 2.73–2.70 (t, 2H, -CH_2_); 2.88 (s, 1H, -CH), 3.79–3.76 (t, 2H, -CH_2_) 7.15–7.13 (d, 1H, Ar-CH_2_); 8.15–8.13 (d, 1H, Ar-CH); ^13^C NMR (100 MHz, CDCl_3_) δ PPM: 24.23, 25.59, 25.70, 26.18, 29.67, 41.59, 116.94, 117.38, 119.01, 120.20, 120.59, 121.59, 128.07, 128.93, 129.15, 132.08, 133.43, 135.77, 151.52, 164.49, 168.56, 176.48; MS (EI) *m*/*z* calculated for C_24_H_25_F_3_N_2_O_3_ 446.47; found 447.46 (M + 1); HPLC purity: 99.06%.

#### 2.2.12. Synthesis of 3,5-Difluoro-N-(1-(tetrahydro-2H-pyran-4-carbonyl)-1,2,3,4-tetrahydroquinolin-7-yl)benzamide (**10c**)

The reaction process is identical to compound **10a**; however, **9c** is reacted with 3,5-difluorobenzoyl chloride in the presence of TEA.

Light yellow crystals yield: 73.77%; mp: 205–207 °C; ^1^H NMR (400 MHz, CDCl_3_) δ PPM: 1.34–1.27 (m, 1H, -CH), 2.05–1.93 (p, 2H, -CH_2_); 2.77–2.74 (t, 2H, -CH_2_), 3.36–3.34 (t, 4H, -CH_2_), 3.60–3.57 (t, 2H, -CH_2_); 3.80–3.65 (m, 4H, -CH_2_), 7.04–6.96 (m, 3H, Ar-CH); 7.08–7.06 (d, 1H, Ar-CH), 7.44–7.37 (m, 1H, Ar-CH), 7.65 (d, 1H, Ar-CH), 8.008 (s, 1H, NH); ^13^C NMR (100 MHz, CDCl_3_) δ PPM: 23.39, 26.59, 45.54, 46.27, 111.50, 111.98, 112.18, 114.65, 124.23, 129.58, 131.89, 131.99, 132.09, 135.93, 140.91, 158.33, 158.61, 151.68, 159.84, 161.12, 161.19; MS (EI) *m*/*z* calculated for C_22_H_22_F_2_N_2_O_3_ 401.41; found 402.42 (M + 1); HPLC purity: 96.35%.

#### 2.2.13. Synthesis of 3-Fluoro-N-(1-(morpholine-4-carbonyl)-1,2,3,4-tetrahydroquinolin-7-yl)-5-(trifluoromethyl)benzamide (**10d**)

The reaction procedure and reagents are the same as those given for **10a**, except **9c** is coupled with 3-fluoro-5-(trifluoromethyl)benzoyl chloride.

White to off white amorphous powder yield: 41.71%; mp: 218–220 °C; ^1^H NMR (400 MHz, CDCl_3_) δ PPM: 1.34–1.27 (m, 1H, -CH), 1.82–1.76 (p, 2H, -CH_2_); 2.64–2.61 (t, 2H, -CH_2_), 3.42–3.40 (t, 4H, -CH_2_), 3.51–3.48 (t, 2H, -CH_2_); 3.71–3.69 t, 4H, -CH_2_), 7.02–7.00 (d, 1H, Ar-CH); 7.26–7.21 (d, 1H, Ar-CH), 7.28 (s, 1H, Ar-CH), 7.31 (s, 1H, Ar-CH), 8.12–810. (m, 1H, Ar-CH), 8.20–8.18 (d, 1H, Ar-CH), 8.88 (s, 1H, NH); ^13^C NMR (100 MHz, CDCl_3_) δ PPM: 23.13, 26.29 29.70, 46.41, 112.59, 115.10, 117.10, 117.36, 118.54, 118.67, 120.88, 123.58, 123.98, 126.92, 129.58, 131.51, 131.54, 133.68, 136.49, 140.34, 160.19, 160.70, 162.78, 163.36 MS (EI) *m*/*z* calculated for C_22_H_21_F_4_N_3_O_3_ 452.41; found 453.42 (M + 1); HPLC purity: 99.35%.

#### 2.2.14. Synthesis of N-(1-(Morpholine-4-carbonyl)-1,2,3,4-tetrahydroquinolin-7-yl)-3,5-bis(trifluoromethyl)benzamide (**10e**)

The reaction procedure and reagents are the same as those given for **10a**, except **9c** is coupled with 3,5-bis(trifluoromethyl)benzoyl chloride.

Yellow amorphous powder yield: 57.67%; mp: 208–209 °C; ^1^H NMR (400 MHz, CDCl_3_) δ PPM: 1.31–1.24 (m, 1H, -CH), 1.48–1.42 (p, 2H, -CH_2_); 2.43–2.39 (t, 2H, -CH_2_), 3.46–3.38 (t t, 6H, -CH_2_), 3.75–3.72 (t, 4H, -CH_2_), 6.98–6.96 (d, 1H, Ar-CH); 7.38–7.28 (d, 1H, Ar-CH), 8.00 (s, 1H, Ar-CH), 8.37 (s, 2H, Ar-CH), 9.61 (s, 1H, NH); ^13^C NMR (100 MHz, CDCl_3_) δ PPM: 14.12, 22.69, 22.79, 25.96, 29.37, 30.18, 31.43, 31.93, 46.58, 46.73, 53.43, 66.58, 113.69, 115.71, 119.00, 121.71, 123.98, 124.54, 127.13, 128.26, 129.53, 131.59, 131.92, 132.25, 136.74, 137.44, 139.81, 161.71, 162.63; MS (EI) *m*/*z* calculated for C_23_H_21_F_6_N_3_O_3_ 501.43; found 502.39 (M + 1); HPLC purity: 97.12%.

#### 2.2.15. Synthesis of 3,5-Difluoro-N-(1-(piperidine-1-carbonyl)-1,2,3,4-tetrahydroquinolin-7-yl)benzamide (**10f**)

The reaction procedure and reagents are the same as those given for **10a**, except **9d** is coupled with 3,5-difluorobenzoyl chloride.

Bright yellow powder yield: 71.20%; mp: 221–223 °C; ^1^H NMR (400 MHz, DMSO-d6) δ PPM: 1.51–1.49 (m, 6H, -CH_2_), 1.87–1.84 (p, 2H, -CH_2_); 2.72–2.68 (t, 2H, -CH_2_), 3.26 (s, 4H, -CH_2_), 3.44–3.41 (t, 2H, -CH_2_), 7.14–7.05 (d, 1H, Ar-CH); 7.25–7.21 (d, 1H, Ar-CH), 7.37–7.36 (t, 2H, Ar-CH), 7.37 (s, 1H, Ar-CH), 7.59–7.54 (p, 1H, Ar-CH), 10.61 (s, 1H, NH); ^13^C NMR (100 MHz, DMSO-d6) δ PPM: 23.03, 24.55, 25.57, 26.50, 46.16, 46.54, 110.24, 112.37, 112.85, 116.06, 122.50, 129.77, 132.28, 132.48, 137.12, 141.49. 157.95, 158.03, 158.30, 159.71, 160.42, 160.50; MS (EI) *m*/*z* calculated for C_22_H_23_F_2_N_3_O_2_ 399.42; found 400.55 (M + 1); HPLC purity: 95%.

#### 2.2.16. Synthesis of 3-Fluoro-N-(1-(piperidine-1-carbonyl)-1,2,3,4-tetrahydroquinolin-7-yl)-5-(trifluoromethyl)benzamide (**10g**)

The reaction procedure and reagents are the same as those given for **10a**, except **9d** is coupled with 3-fluoro-5-(trifluoromethyl)benzoyl chloride.

Off white powder yield: 44.32%; mp: 213–215 °C; ^1^H NMR (400 MHz, CDCl_3_) δ PPM: 1.63–1.59 (m, 6H, -CH_2_), 1.74–1.71 (p, 2H, -CH_2_); 2.61–2.59 (t, 2H, -CH_2_), 3.37–3.36 (s, 4H, -CH_2_), 3.44–3.42 (t, 2H, -CH_2_), 6.98–6.96 (d, 1H, Ar-CH); 7.15 (d, 1H, Ar-CH), 7.36–7.24 (m, 1H, Ar-CH), 7.38–7.36 (d, 1H, Ar-CH), 8.15–8.12 (m, 1H, Ar-CH), 8.24–8.22 (d, 1H, Ar-H), 9.18 (s, 1H, NH); ^13^C NMR (100 MHz, CDCl_3_) δ PPM: 23.34, 24.56, 25.12, 26.35, 46.22, 46.56, 110.35, 112.43, 112.54, 116.78, 122.23, 129.65, 132.28, 132.48, 137.12, 141.49. 157.95, 158.03, 158.30, 159.71, 160.42, 160.50; MS (EI) *m*/*z* calculated for C_23_H_23_F_4_N_3_O_2_ 449.45; found 450.53 (M + 1); HPLC purity: 99.36%.

#### 2.2.17. Synthesis of N-(1-(Piperidine-1-carbonyl)-1,2,3,4-tetrahydroquinolin-7-yl)-3,5 bis(trifluoromethyl)benzamide (**10h**)

The reaction procedure and reagents are the same as those given for **10a**, except **9d** is coupled with 3-5-(trifluoromethyl)benzoyl chloride.

Yellow powder yield: 65.23%; mp: 207–208 °C; ^1^H NMR (400 MHz, CDCl_3_) δ PPM: 1.30–1.27 (s, 2H, CH_2_); 1.54–1.51 (p, 2H, -CH_2_); 1.67–1.63 (m, 6H, -CH_2_), 2.51–2.47 (t, 2H, -CH_2_), 3.42–3.40 (m, 6H, -CH_2_), 7.00–6.98 (d, 1H, Ar-CH); 7.18 (d, 1H, Ar-CH), 7.43–7.40 (dd, 1H, Ar-CH), 8.01 (s, 1H, Ar-CH), 8.24–8.22 (d, 1H, Ar-H), 8.39 (s, 2H, Ar-CH), 9.33 (s, 1H, NH); ^13^C NMR (100 MHz, CDCl_3_) δ PPM: 22.65, 24.60, 25.85, 26.06 29.07, 30.17 31.43, 47.15, 113.63, 115.32, 121.75, 123.76, 124.59, 124.76, 127.18, 128.41, 129.44, 131.17, 131.51, 132.17, 136.73, 137.64, 140.16, 161.99, 162.77; MS (EI) *m*/*z* calculated for C_24_H_23_F_6_N_3_O_2_ 449.46; found 500.52 (M + 1); HPLC purity: 97.18%.

### 2.3. Evaluation of Antiproliferative Activity by MTT Assay

To evaluate the antiproliferative activity of the synthesized tetrahydroquinoline derivatives, three distinct human cancer cell lines were selected: breast cancer (MCF-7), lung cancer (A-549), and triple-negative breast cancer (MDA-MB-231) [39,40,41]. Furthermore, the synthesized compounds were tested on the VERO cell line to assess their potential toxicity. The VERO cell line, commonly derived from the kidney of an adult African green monkey, is widely used for toxicity studies [42]. To evaluate the toxicity of the synthesized compounds, this cell line was utilized in the present study. The VERO cells were cultured using RPMI-1650 medium [43]. The F-12 Ham’s medium was used to cultivate the lung cancer (A-549) cell lines [44]. DMEM was used to cultivate the cell lines for breast cancer (MCF-7) and triple-negative breast cancer (MDA-MB-231). Because it feeds the cell line, 5% fatal bovine serum (FBS) was used to make the whole medium. To further protect the cell line from contamination, 0.5% pen–strep (penicillin + streptomycin) was added to all the mediums. All of these cell lines were cultivated in sterile, vented T-75 and/or T-25 flasks that were kept at 37 °C and supplied with 5% CO_2_. All of the compounds’ stock solutions were prepared in DMSO, and dilutions were made in the appropriate media so that the 96-well plates’ final DMSO content was not greater than 0.1%. First, each drug was tested in triplicate against every cell line at a 25 µM dose. To prepare the serial dilutions and calculate the IC_50_ values, the compounds exhibiting greater than 50% inhibition were selected. On the first day, 1 × 104 cells were planted, and 24 h later, the chemicals were added. The MTT dye was added after the compounds and cells had been cultured for 48 h. After four hours, the MTT dye was taken out of the wells. Each well received 100 µL of DMSO to dissolve the formazan crystals that had formed as a result of the living cells, and absorbance at 570 nm was measured [45].

### 2.4. Cytotoxic Effects Leading to Apoptosis

A549 lung cancer cells were seeded in six-well plates and cultured in F-12 Ham’s medium supplemented with 5% FBS, 0.5% penicillin–streptomycin, and maintained in a 5% CO_2_ atmosphere at 37 °C for 24 h. The three most potent compounds, based on IC_50_ values, were selected and tested at concentrations of 3 µM and 6 µM. Each condition was maintained in duplicate, with DMSO serving as the vehicle control, while 5-fluorouracil and Everolimus were used as positive controls. The treated cells, along with the DMSO control group, were incubated for 48 h. After incubation, the media were removed, and 1 mL of trypsin was added to each well for approximately one minute, followed by neutralization with 4 mL of fresh medium. Subsequent steps were carried out following the protocol of the Annexin V-FITC apoptosis detection kit (Ref No: BMS500FI, Lot No: 264043; Invitrogen, Waltham, MA, USA). The contents of each well were collected and transferred to centrifuge tubes, followed by centrifugation at 1000 RPM for 8 min. After removing the supernatant, the cell pellets were washed with PBS and subjected to another centrifugation step. The pellets were then resuspended in 1 mL of 1× binding buffer from the Annexin V-FITC apoptosis detection kit and incubated in the dark for 15–20 min. Subsequently, 500 µL of this cell suspension was transferred to individual plastic flow cytometry tubes. Each tube was supplemented with 10 µL of propidium iodide (PI) and 5 µL of Annexin V-FITC conjugate. The tubes were then incubated in the dark for 10 min before analyzing cell apoptosis using flow cytometry [46,47,48].

### 2.5. Molecular Docking

Docking studies were performed using AutoDock Vina software version 4.2.6 [49,50]. The 3D structure of the core protein mTOR (PDB ID: 4JT6) was obtained from the Protein Data Bank (https://www.rcsb.org/ accessed on 08 January 2025). The water molecules and native ligands were removed using a Discovery Studio Visualizer v 2021. Then, the protein was imported into the Autodock tool 4.2.6 for the preparation process, including hydrogenation and charge modification. This resulted in the modified target protein and the result protein bring exported as PDBQT files. All ligands were also exported as PDBQT files. Docking simulations were conducted using Auto Dock Vina software version 1.2.0. The X, Y, and Z coordinates for mTOR protein were kept at −17.403, −33.444, and −54.547, respectively, and the dimensions of the grid were kept at 58, 58, and 44 Å, respectively. The docking results were analyzed and visualized using Discovery Studio software v21.1.0.20298.

### 2.6. Molecular Dynamics Simulation Study

Molecular dynamics (MD) simulation was conducted using GROMACS 2021.4 (https://manual.gromacs.org/2021.4/index.html accessed on 14 January 2025) to model the dynamic interactions between the protein and ligand, thereby validating the accuracy of the drug design and supporting the study’s rationale [51,52,53]. Protein topology files were generated with the OPLS force field (Optimized Potential for Liquid Simulations, version 15) using the ’pdb2gmx’ command. Ligand files compatible with GROMACS were prepared via the LigParagen web server (https://traken.chem.yale.edu/ligpargen/ accessed on 14 January 2025). The protein was immersed in a dodecahedral simulation box using a three-point water model and the charge was neutralized by adding four Na^+^ ions, and periodic boundary conditions were applied in all directions to eliminate edge effects. The minimization of energy in the protein-ligand system was performed using the ’gmx grompp’ and ’gmx mdrun’ commands. Equilibration was achieved under NVT (canonical) and NPT (isobaric) ensembles, with a V-rescale thermostat maintaining a constant temperature of 300 K and a fixed volume. The system underwent a 100 ns MD simulation, during which stability analyses were conducted. These included calculations of the root mean square deviation (RMSD), root mean square fluctuation (RMSF), radius of gyration (Rg), solvent-accessible surface area (SASA), and hydrogen bond count (HBond) using the ’gmx rms’ command.

## 3. Results and Discussion

### 3.1. Chemistry

A strategic synthetic approach was used to synthesize eight new tetrahydroquinoline (THQ) derivatives, as shown in Figure 2. To protect the THQ molecule’s nitrogen atom, 9-fluorenemethoxycarbonyl (Fmoc) was first applied to it. When THQ reacted with sulfuric acid and potassium nitrate, this protection allowed for selective nitration at the seventh position. Pyrrolidine was used to deprotect the Fmoc-protected THQ after nitration. The resulting compound was then reacted with various acid chlorides, including tetrahydro-2H-pyran-4-carbonyl chloride, cyclohexanecarbonyl chloride, morpholine-4-carbonyl chloride, and piperidine-1-carbonyl chloride, as outlined in the synthetic scheme. Subsequently, these intermediates were treated with ammonium chloride and zinc to reduce the nitro group to an amine. The resulting primary amine was then subjected to reactions with different aromatic acid chlorides (denoted as R in the scheme), culminating in the synthesis of the desired eight novel THQ derivatives. The final compounds were characterized using ^1^H and ^13^C NMR spectroscopy and mass spectrometry, and their purity was assessed via HPLC analysis. All synthesized derivatives exhibited >95% purity, as confirmed by the HPLC data. Detailed spectra and chromatograms are provided in the Supplementary Materials section.

### 3.2. In Vitro Antiproliferative MTT Assay

The MTT assay results revealed that morpholine-substituted tetrahydroquinoline derivatives exhibited potent and selective cytotoxicity against A549, MCF-7, and MDA-MB-231 cancer cell lines, with minimal toxicity towards Vero cells. Compound **10e** demonstrated the highest activity against A549 cells (IC_50_ = 0.033 ± 0.003 µM), while **10h** was most effective against MCF-7 cells (IC_50_ = 0.087 ± 0.007 µM). Compound **10d** showed broad-spectrum activity with IC_50_ values of 0.062 ± 0.01 µM, 0.58 ± 0.11 µM, and 1.003 ± 0.008 µM for A549, MCF-7, and MDA-MB-231, respectively. Structure–activity relationship analysis indicated that compounds with X = O and Y = N (e.g., **10d** and **10e**) exhibited superior activity compared to those with X = CH_2_ or Y = CH, likely due to enhanced mTOR interaction facilitated by the electronegative oxygen.

### 3.3. Structure–Activity Relationship

Structure–activity relationship (SAR) research on the morpholine-substituted tetrahydroquinoline derivatives provides important information about how certain structural characteristics and functional groups affect the cytotoxic activity against MDA-MB-231, MCF-7, and A549 cancer cell lines. The cytotoxic action is greatly influenced by the substituents on the benzamide molecule. Two trifluoromethyl groups that are highly electron-withdrawing increased the cytotoxicity of all investigated cell lines. With these groups, compound **10e** was the most potent derivative, showing the lowest IC_50_ values for MDA-MB-231 (0.63 ± 0.02 µM) and A549 (0.033 ± 0.003 µM). Similarly, **10h**, which also features 3,5-bis(trifluoromethyl) substitution, showed excellent activity against MCF-7 cells (IC_50_ = 0.087 ± 0.007 µM) illustrated in Table 1. The potency of these derivatives can be attributed to enhanced interactions with the mTOR active site, likely through halogen bonding and hydrophobic interactions mediated by the trifluoromethyl groups. The combination of a single fluorine atom at position 3 and a trifluoromethyl group at position 5 also resulted in high activity. Compound **10d** displayed IC_50_ values of 0.062 ± 0.01 µM for A549 and 0.58 ± 0.11 µM for MCF-7. This suggests that the strategic placement of fluorine and trifluoromethyl groups is optimal for maintaining high activity while reducing toxicity. Substitution with two fluorine atoms at positions 3 and 5 resulted in moderate activity. Compound **10c** showed IC_50_ values of 3.73 ± 0.17 µM across the lung cancer cell lines, while compound **10f** displayed selective activity against MCF-7 (IC_50_ = 4.47 ± 0.013 µM). The decreased potency compared to trifluoromethyl derivatives suggests that the bulkier and more electronegative trifluoromethyl groups provide stronger interactions and better activity.

**Figure 3:**
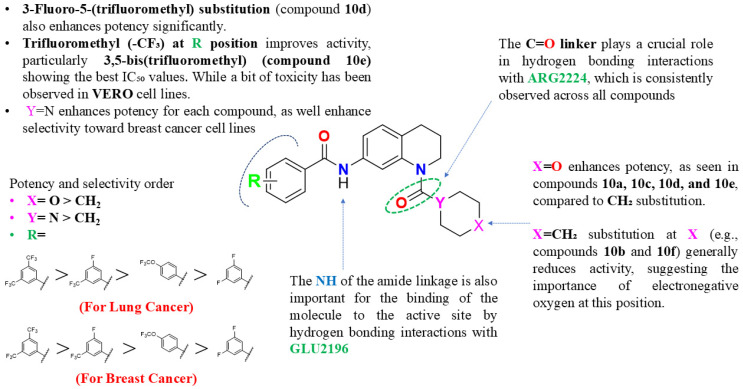
SAR insights: effect of functional groups on the activity of synthesized THQ derivatives.

The oxygen atom (X = O) plays a pivotal role in enhancing cytotoxic activity. Electronegative oxygen enhances the interaction potential of the molecule with mTOR binding residues, possibly by forming hydrogen bonds or improving electronic complementarity. Compounds with X = O consistently outperformed their counterparts with X = CH_2_ in terms of potency. For example, **10e** (X = O) was significantly more active than **10h** (X = CH_2_), despite having identical R and Y groups. Replacing the oxygen with a methylene group reduced activity across all cell lines. For instance, **10f** (X = CH_2_, R = 3,5-difluoro) exhibited no activity against A549 or MDA-MB-231, in contrast to its X = O counterpart, **10c**, which showed moderate activity. This highlights the importance of the oxygen atom in maintaining mTOR binding interactions. The nitrogen atom enhances the electronic complementarity of the molecule and contributes to better binding with mTOR residues. Compounds with Y = N consistently showed higher activity compared to their Y = CH counterparts. For instance, **10a** (Y = CH, X = O) had an IC_50_ of 1.06 ± 0.02 µM against A549, whereas **10c** (Y = N, X = O) displayed improved potency with an IC_50_ of 3.73 ± 0.17 µM across all cancer lines. All derivatives exhibited minimal cytotoxicity towards Vero cells (IC_50_ >25 µM for most compounds), indicating good selectivity for cancer cells. Compounds with strong electron-withdrawing substituents (e.g., **10e** and **10d**) showed the best selectivity index (SI), demonstrating their potential as targeted mTOR inhibitors, as described in Figure 3.

### 3.4. Apoptosis Assay

**Figure 4:**
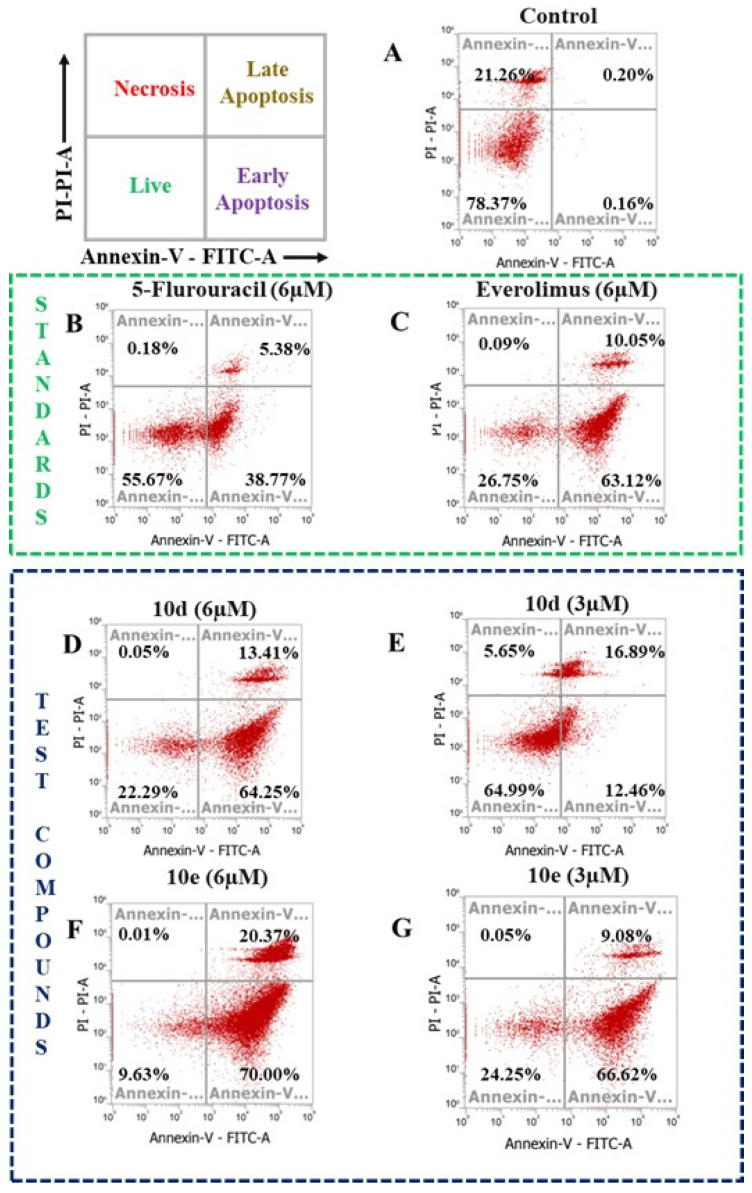
(**A**) DMSO control cells of lung cancer (A549); (**B**) effect of 5-FU at 6 µM; (**C**) effect of Everolimus at 6 µM; (**D**) effect of compound **10d** at 6 µM; (**E**) effect of compound **10d** at 3 µM; (**F**) effect of compound 10d at 6µM; (**G**) effect of compound **10d** at 3 µM.

The FACS analysis results, illustrated in Figure 4A–G, provide a detailed examination of the apoptotic potential of the studied compounds, including the controlled (Figure 4A) the standard chemotherapeutic agents 5-FU (Figure 4B) and Everolimus (Figure 4C), alongside four novel derivatives. This characterization offers critical insights into their mechanisms of action, apoptotic pathways, and potential as therapeutic agents.

Treatments of A549 cells with the synthesized compounds **10d** and **10e** at the concentrations of 6 µM (Figure 4D) and 3 µM (Figure 4E) for 48 h demonstrated a significant induction of apoptosis (early and last phase combined). Specifically, compound **10d** induced 29.35% apoptosis at 3 µM and 77.66% at 6 µM (Figure 4F,G), while compound **10e** exhibited even greater efficacy, with apoptosis levels reaching 75.7% and 90.37% at the same concentrations. In contrast, the control group treated with DMSO showed only 0.36% apoptosis in 44.15% of cells, serving as a comparative benchmark. A closer examination of the effects of **10e** at 6 µM revealed profound changes in cell viability and apoptotic induction. The percentage of live cells decreased drastically from 78.37% (Figure 4A) in the control group to 24.25% (Figure 4G) in the treated group. This reduction in cell viability was accompanied by a sharp increase in apoptotic cells, rising from a negligible 0.36% in the control group to an impressive 90.37%. Notably, the apoptotic population consisted of 70.00% early apoptotic cells and 20.37% late apoptotic cells, indicating that compound **10e** primarily drives cells into the early stages of programmed cell death. This mode of action suggests a targeted apoptotic mechanism rather than necrosis, as evidenced by the negligible necrotic cell population observed (0.05% at 3 µM and 0.01% at 6 µM).

The comparison with 5-FU highlights the remarkable efficacy of compound **10e**. While 5-FU is a well-established chemotherapeutic agent, **10e** suggests a more controlled Sand potentially less inflammatory cell death pathway, which is a desirable characteristic in anticancer agents to minimize adverse effects on surrounding tissues. The observed apoptotic effects of compounds **10d** and **10e** were dose-dependent, with higher concentrations yielding greater efficacy. This dose–response relationship emphasizes the potential for fine-tuning therapeutic regimens to maximize efficacy while minimizing side effects. Additionally, the significant early apoptotic activity observed with **10e** could be explored further to understand its impact on downstream apoptotic signaling pathways, such as caspase activation and mitochondrial membrane potential disruption.

This comprehensive analysis not only underscores the potent apoptotic properties of compound **10e** but also establishes its potential as a hit compound for the development of novel anticancer therapies. The findings pave the way for future studies, including mechanistic investigations into its molecular targets, in vivo efficacy in animal models, and optimization of its pharmacokinetic and pharmacodynamic profiles. With its demonstrated apoptotic potency, minimal necrosis, and favorable comparison to 5-FU, compound **10e** represents a promising candidate for clinical development, offering hope for improved therapeutic options in the treatment of lung cancer.

### 3.5. Molecular Docking Studies

**Figure 5:**
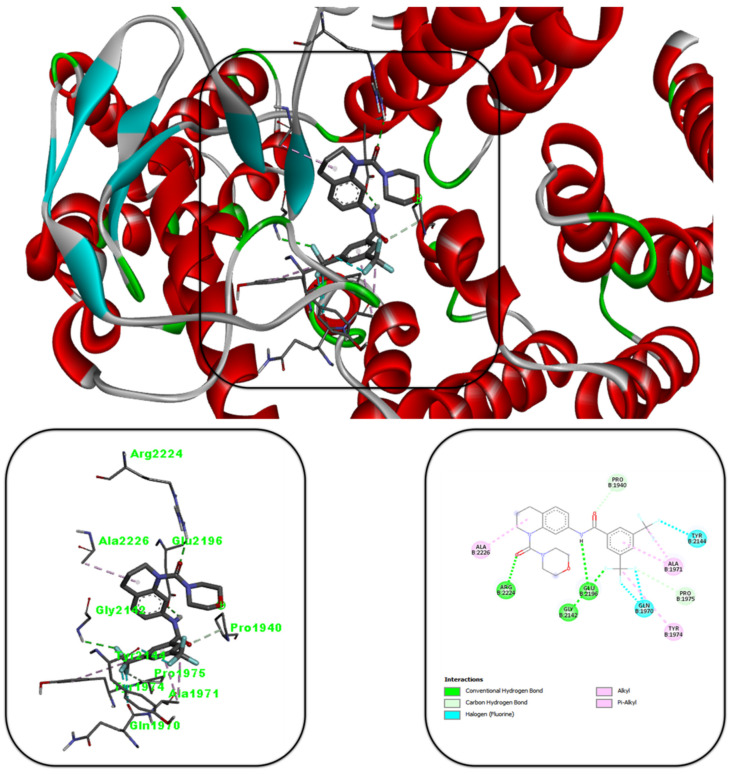
Molecular docking view of compound **10e** in the 4JT6 binding site.

To explore the potential mode of action, binding mode analysis was conducted for all the synthetic compounds, with compound **10e** demonstrating the most favorable results compared to the co-crystal ligand (X6K) as presented in Figure 5 and Figure S2 of the supplementary file. The docking score of compound **10e** (−10.6 kcal/mol) indicated a lower binding energy than the co-crystal ligand (−10.1 kcal/mol), highlighting its enhanced binding efficiency. The binding interactions of compound **10e** are summarized in Figure 5. The carbonyl group (C=O) forms a hydrogen bond with Arg2224, while the fluorine group establishes a hydrogen bond with Glu2142. Additionally, the -NH group forms a hydrogen bond with Glu2196. These interactions suggest that the synthesized compound binds stably to the mTOR protein and exhibits cytotoxic effects.

### 3.6. MD Simulation

**Figure 6:**
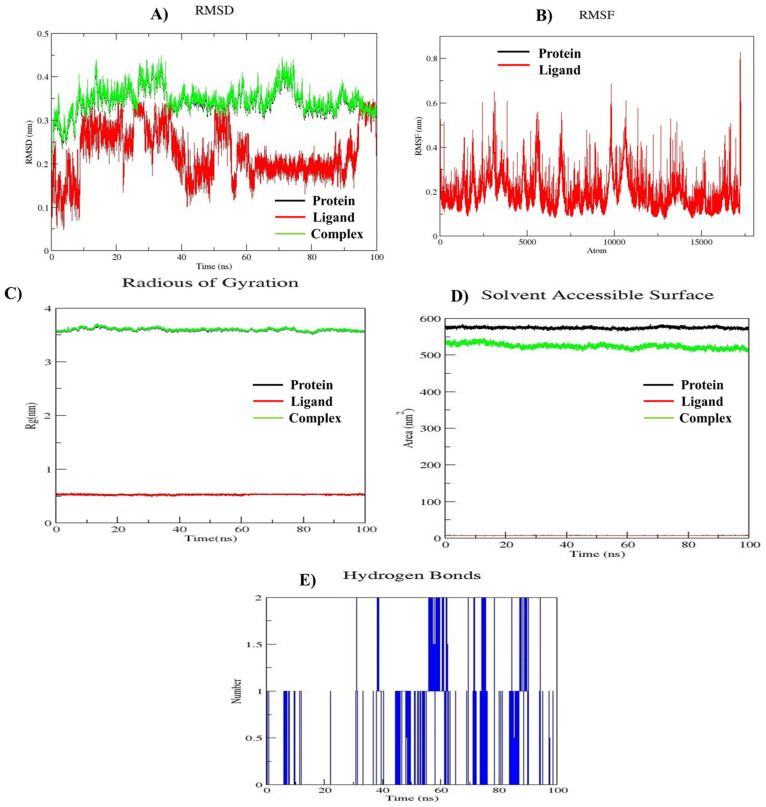
Plots of MD simulations of the complex of compound **10e** with mTOR protein for 100 ns. (**A**) RMSD; (**B**) RMSF; (**C**) Rg; (**D**) SASA plot; (**E**) number of hydrogen bonds.

The molecular dynamics simulation results for the mTOR protein (PDB ID: 4JT6) in complex with compound **10e**, as depicted in Figure 6, demonstrate the stability of the protein–ligand complex throughout the 100 ns simulation period. This is supported by the root mean square deviation (RMSD) graph Figure 6A, which shows consistent stability over time. The root mean square fluctuation (RMSF) analysis, shown in Figure 6B, reveals moderate fluctuations ranging from 0.1 nm to 0.8 nm. This indicates dynamic behavior of residues at the protein–ligand interface while maintaining overall stability. Additionally, the compactness and structural integrity of the complex are confirmed by the radius of gyration (Rg) values as shown in Figure 6C, which remain close to 3.5 nm. The solvent-accessible surface area (SASA), as illustrated in Figure 6D, highlights stable folding and compactness of the protein structure, further confirming the system’s stability throughout the simulation. Hydrogen bonds, which play a critical role in the complex’s stability and rigidity, were also analyzed. As shown in Figure 6E, the hydrogen bonds formed between the ligand and the protein persist consistently, restricting protein movement and enhancing stability. These results collectively indicate that the mTOR-compound **10e** complex is structurally stable and dynamically active, offering valuable insights into its potential as a therapeutic target. These findings warrant further investigation and support its promise in drug development efforts.

## 4. Discussion

This study successfully demonstrates the synthesis, characterization, and evaluation of morpholine-substituted tetrahydroquinoline (THQ) derivatives as potential mTOR inhibitors. The strategic inclusion of morpholine and trifluoromethyl groups in the THQ scaffold was pivotal in achieving enhanced selectivity and potency against cancer cell lines. Molecular docking and dynamics simulations corroborated the in vitro results, establishing the compounds’ potential as therapeutic agents for targeting mTOR in lung cancer.

The synthetic strategy yielded eight novel THQ derivatives, with each compound characterized by spectroscopic techniques, including ^1^H NMR, ^13^C NMR, and mass spectrometry. High-performance liquid chromatography (HPLC) confirmed the purity of all derivatives (>95%). This meticulous characterization ensured the structural integrity and quality of the compounds, which is critical for correlating their structural features with biological activity.

The MTT assay results highlighted the significant antiproliferative activity of the synthesized compounds against A549, MCF-7, and MDA-MB-231 cancer cell lines. Compound **10e** emerged as the most potent derivative, exhibiting an IC_50_ value of 0.033 ± 0.003 µM against A549 cells. The structure–activity relationship (SAR) analysis revealed that electronegative substituents, such as trifluoromethyl groups, significantly enhance binding affinity to the mTOR active site. Additionally, the inclusion of oxygen (X = O) in the scaffold improved cytotoxic activity, emphasizing the importance of electronic and steric factors in mTOR inhibition.

Flow cytometry results demonstrated that compounds **10d** and **10e** induced apoptosis in a dose-dependent manner, with compound **10e** achieving over 90% apoptosis at 6 µM in A549 cells. The predominance of early apoptosis suggests a specific and controlled mechanism of action, distinguishing these derivatives from necrosis-inducing agents. This targeted apoptotic effect aligns with the desired therapeutic profile for anticancer agents, minimizing off-target effects.

Molecular docking studies revealed strong binding interactions between the derivatives and key residues in the mTOR active site, such as Arg2224 and Glu2142. The docking score of compound **10e** (−10.6 kcal/mol) surpassed that of the co-crystal ligand (−10.1 kcal/mol), indicating superior binding efficiency. Molecular dynamics simulations further validated these findings, showing stable protein–ligand interactions over a 100 ns simulation period. Parameters, such as root mean square deviation (RMSD), radius of gyration (Rg), and hydrogen bonding, confirmed the structural stability of the complexes.

## 5. Conclusions

This research underscores the potential of morpholine-substituted tetrahydroquinoline derivatives as promising mTOR inhibitors for targeted cancer therapy, particularly against lung cancer. Among the synthesized compounds, **10e** exhibited the most potent cytotoxic activity against A549 cells, with an exceptional IC_50_ value of 0.033 ± 0.003 µM, highlighting its potential as a lead candidate. Compound **10h** demonstrated remarkable activity against MCF-7 cells (IC_50_ = 0.087 ± 0.007 µM), and **10d** showed broad-spectrum efficacy across A549, MCF-7, and MDA-MB-231 cells. These derivatives not only exhibited high selectivity indices but also demonstrated a dose-dependent induction of apoptosis, particularly through early apoptotic pathways, thereby minimizing necrotic and off-target effects.

The molecular docking and dynamics simulations further confirmed the experimental observations. Compound **10e**, with a docking score of −10.6 kcal/mol, demonstrated superior binding efficiency and stability within the mTOR active site compared to the co-crystal ligand [54]. The structural integrity and stability of the protein–ligand complexes over the simulation period reinforce the therapeutic promise of these derivatives.

These findings advocate the need for further preclinical evaluations, including in vivo efficacy studies and pharmacokinetic profiling. The incorporation of trifluoromethyl groups and morpholine moieties in the THQ scaffold emerges as a critical design strategy for developing effective and selective mTOR inhibitors. Compound **10e**, in particular, represents a compelling candidate for advancing lung cancer therapeutics.

## Figures and Tables

**Table 1:** Compounds along with their IC_50_ values in different cell lines.

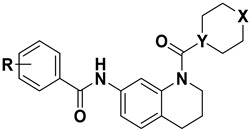								
Sl No.	Compound No.	X	Y	R	A549 (IC_50_ µM)	MCF-7 (IC_50_ µM)	MDA-MB-231 (IC_50_ µM)	VERO (IC_50_ µM)
1	5-FU	**-**	**-**	**-**	0.28 ± 0.008	0.72 ± 0.03	3.39 ± 0.37	>25
2	Everolimus	**-**	**-**	**-**	0.09 ± 0.01	5.86 ± 0.07	7.76 ± 0.37	>25
3	**10a**	O	CH	4-(trifluoromethoxy)	1.06 ± 0.02	4.34 ± 0.12	8.16 ± 0.33	>25
4	**10b**	CH_2_	CH	4-(trifluoromethoxy)	4.72 ± 0.11	>25	6.37 ± 0.19	9.82 ± 0.08
5	**10c**	O	N	3,5-difluoro	3.73 ± 0.17	8.31 ± 0.43	>25	>25
6	**10d**	O	N	3-fluoro-5-(trifluoromethyl)	0.062 ± 0.01	0.58 ± 0.11	1.003 ± 0.008	>25
7	**10e**	O	N	3,5-bis(trifluoromethyl)	0.033 ± 0.003	2.89 ± 0.013	0.63 ± 0.02	8.86 ± 0.03
8	**10f**	CH_2_	N	3,5-difluoro	>25	4.47 ± 0.013	>25	>25
9	**10g**	CH_2_	N	3-fluoro-5-(trifluoromethyl)	0.68 ± 0.13	2.50 ± 0.16	>25	>25
10	**10h**	CH_2_	N	3,5-bis(trifluoromethyl)	3.36 ± 0.71	0.087 ± 0.007	1.29 ± 0.032	>25

## Data Availability

The data presented in this study are available at a reasonable request from the corresponding authors.

## Supplementary Materials

The following supporting information can be downloaded at: https://www.mdpi.com/article/10.3390/cancers17050759/s1, Figure S1: HPLC chromatogram of the mixture of compounds, **10 a–h**: Spectral characterization and HPLC chromatogram of the synthesized compounds, Figure S2: Ligand interaction diagram with the co-crystal ligand X6K (2D and 3D).
