# Supplementary material for: Morpholine-Substituted Tetrahydroquinoline Derivatives as Potential mTOR Inhibitors: Synthesis, Computational Insights, and Cellular Analysis
Source: Cancers (Basel). 2025 Feb 23;17(5):759. doi: 10.3390/cancers17050759 (PMC11898650; doi:10.3390/cancers17050759)
Supplement: Supplementary file 1 [file cancers-17-00759-s001.zip › cancers-3472759-supplementary.pdf]

Morpholine-Substituted Tetrahydroquinoline Derivatives as Potential mTOR Inhibitors: Synthesis, Computational Insights, and Cellular Analysis

Rajdeep Dey [a]; Suman Shaw [a]; Ruchi Yadav [b]; Bhumika Patel [a]; Hardik Bhatt [a]; Gopal Natesan [a]; Abhishek Jha [c]*; Udit Chaube [a]*

[a] Department of Pharmaceutical Chemistry, Institute of Pharmacy, Nirma University Ahmedabad, 382481, Gujarat, India

[b] Department of Pharmacology, Institute of Pharmacy, Nirma University Ahmedabad, 382481, Gujarat, India

[c] Department of Internal Medicine, Roy J.and Lucille A. Carver College of Medicine, University of IOWA, Iowa City, IA 52242, United States of America

Rajdeep Dey and Suman Shaw contributed equally to the study.

[a*]Corresponding Author

Department of Pharmaceutical Chemistry, Institute of Pharmacy, Nirma University, Ahmedabad, 382481, Gujarat, India

Email: uditchoube@gmail.com, udit.chaube@nirmauni.ac.in (Institutional Email ID)

Ph.+91-79-71652718

Fax +91-2717-241916

[c*]Corresponding Author

Department of Internal Medicine, Roy J. and Lucille A. Carver College of Medicine, University of IOWA, Iowa City, IA 52242, United States of America. abhishek-jha@uiowa.edu

SL No	Figure/ Table No	Figure/ Table Caption	Page No
**1**	SF-01	HPLC chromatogram of the mixture of compounds	1
**2**	10 a	Spectral Characterization of the synthesized compounds	2-5
**3**	10 b	Spectral Characterization of the synthesized compounds	6-9
**4**	10 c	Spectral Characterization of the synthesized compounds	10-13
**5**	10 d	Spectral Characterization of the synthesized compounds	14-17
**6**	10 e	Spectral Characterization of the synthesized compounds	18-21
**7**	10 f	Spectral Characterization of the synthesized compounds	22-25
**8**	10 g	Spectral Characterization of the synthesized compounds	26-29
**9**	10 h	Spectral Characterization of the synthesized compounds	30-33
**10**	SF-02	Ligand interaction diagram with the co-crystal ligand X6K (2D & 3D)	34

Sample Name: NitroTHQ

Acquired by	: Admin
Date Acquired	: 2/11/2024 9:20:46 PM
Sample ID	: 12
Vial#	: 12
Injection Volume	: 10
Data File	: NitroTHQ_.lcd
Method file	:250_SUNFIRE_NORMAL METHOD.lcm

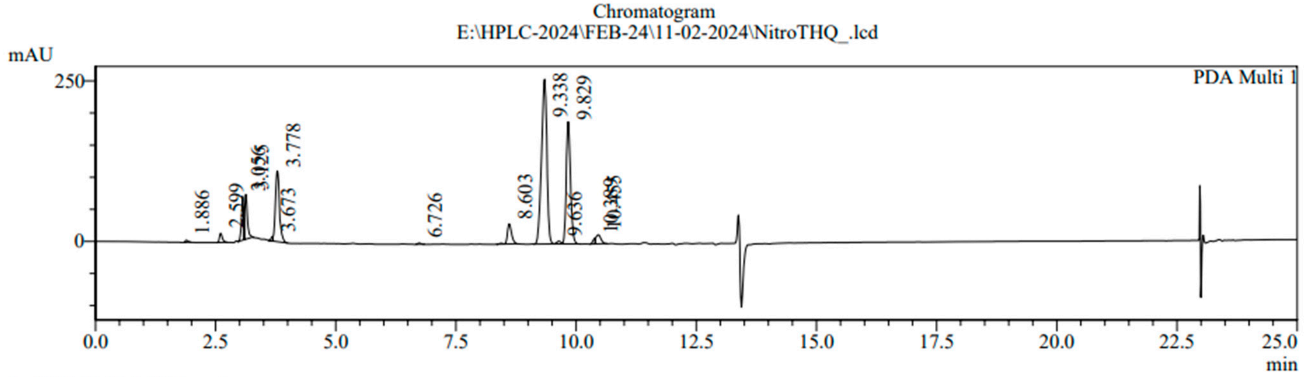

1 PDA Multi 1 / 261nm 4nm

PeakTable

PDA Ch1 261nm 4nm

Peak#	Ret. Time	Area	Height	Area %
1	1.886	11598	3333	0.245
2	2.599	59074	14294	1.248
3	3.056	218612	68450	4.617
4	3.125	262373	69862	5.541
5	3.673	19137	6644	0.404
6	3.778	635455	109978	13.420
7	6.726	8669	1780	0.183
8	8.603	195498	31575	4.129
9	9.338	1976386	256791	41.738
10	9.636	28385	4432	0.599
11	9.829	1193042	190636	25.195
12	10.389	31293	8770	0.661
13	10.455	95682	14119	2.021
Total		4735205	780663	100.000

Supplementary Figure S1: HPLC chromatogram of the mixture of compounds (Not Purified)

Spectral Characterization of the synthesized compounds (10 a-h)

1.1. Mass spectra of compound 10a

S A M P L E   I N F O R M A T I O N

Sample Name:	UC-RS-07	Acquired By:	System
Sample Type:	Unknown	Date Acquired:	10-08-2024 15:23:48 IST
Vial:	1:A,5	Acq. Method Set:	ARAL_MASS_
Injection #:	1	Date Processed:	10-08-2024 15:36:21 IST, 10-08-2024
Injection Volume:	2.00 ul	Processing Methoc	Aral_Mass_
Run Time:	1.5 Minutes	Channel Name:	MS TIC
Sample Set Name	10082024_MASS	Proc. Chnl. Descr.	SQ 2: MS Scan MS TIC (2:

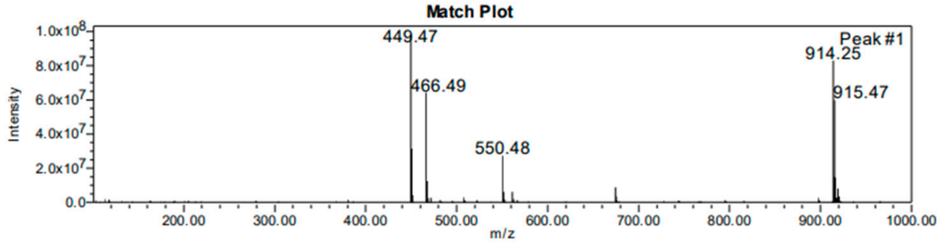

SampleName: UC-RS-07 Injection: 1 Name: Match1 Threshold: Base Peak 449.47 Channel Type 3D MS Channel Description 2: 100.00-1000.00 ES+, Centroid, CV=30

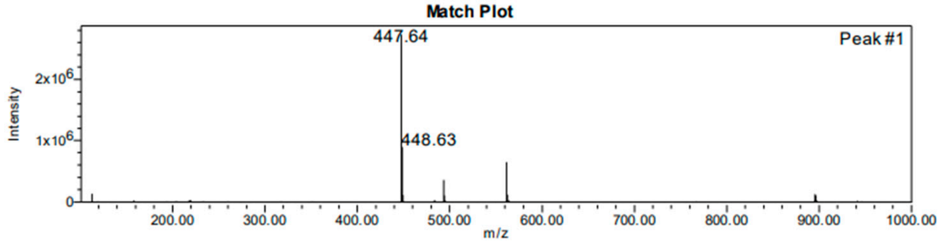

SampleName: UC-RS-07 Injection: 1 Name: Match1 Threshold: Base Peak 447.64 Channel Type 3D MS Channel Description 5: 100.00-1000.00 ES-, Centroid, CV=50

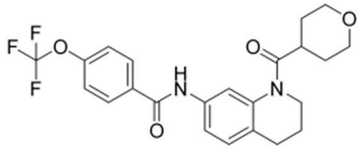

Exact Mass: 448.16
Molecular Weight: 448.44

1.2. ^{1}H NMR of compound 10a

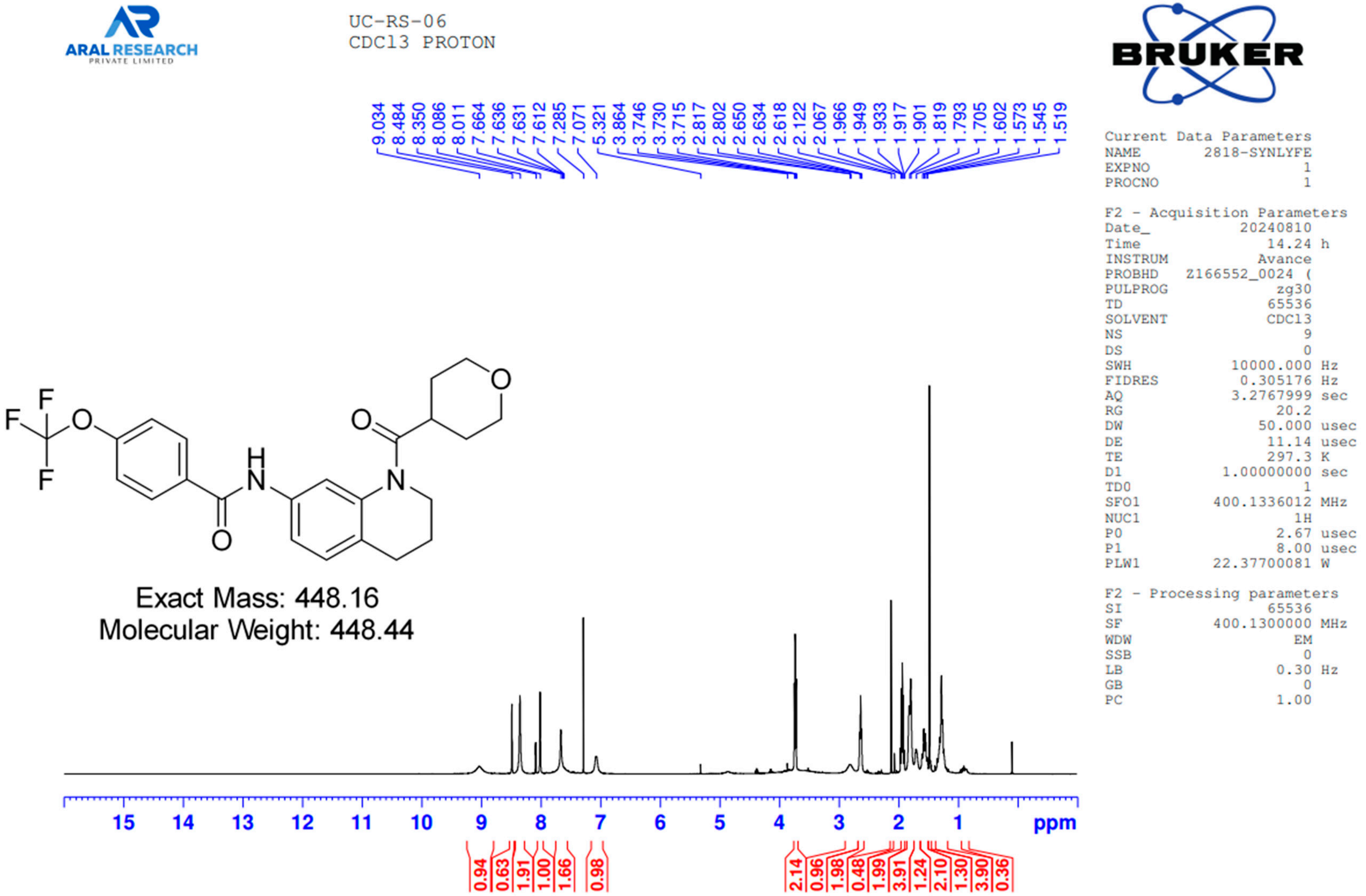

1.3. ^{13}C NMR of compound 10a

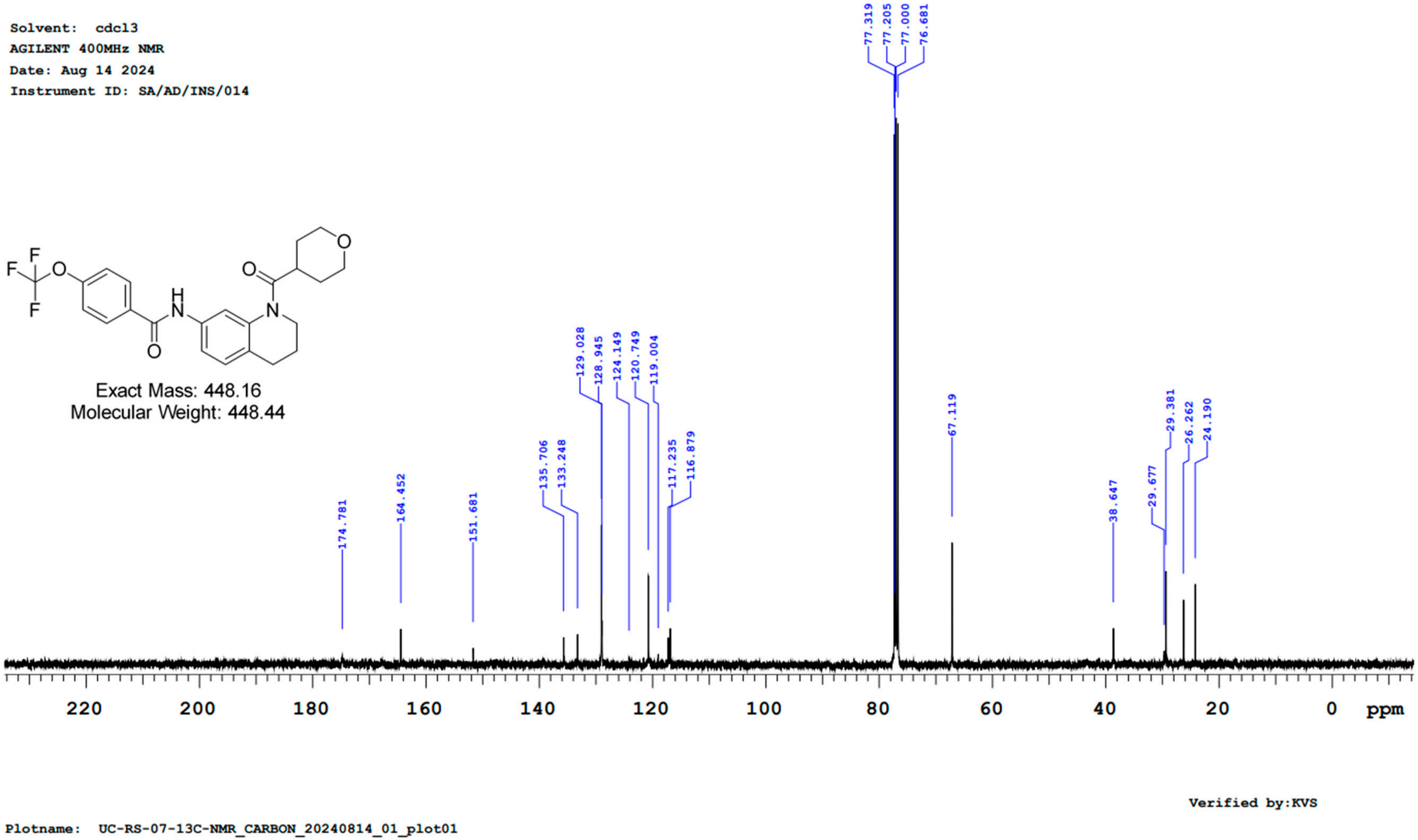

1.4. HPLC chromatogram of compound 10a

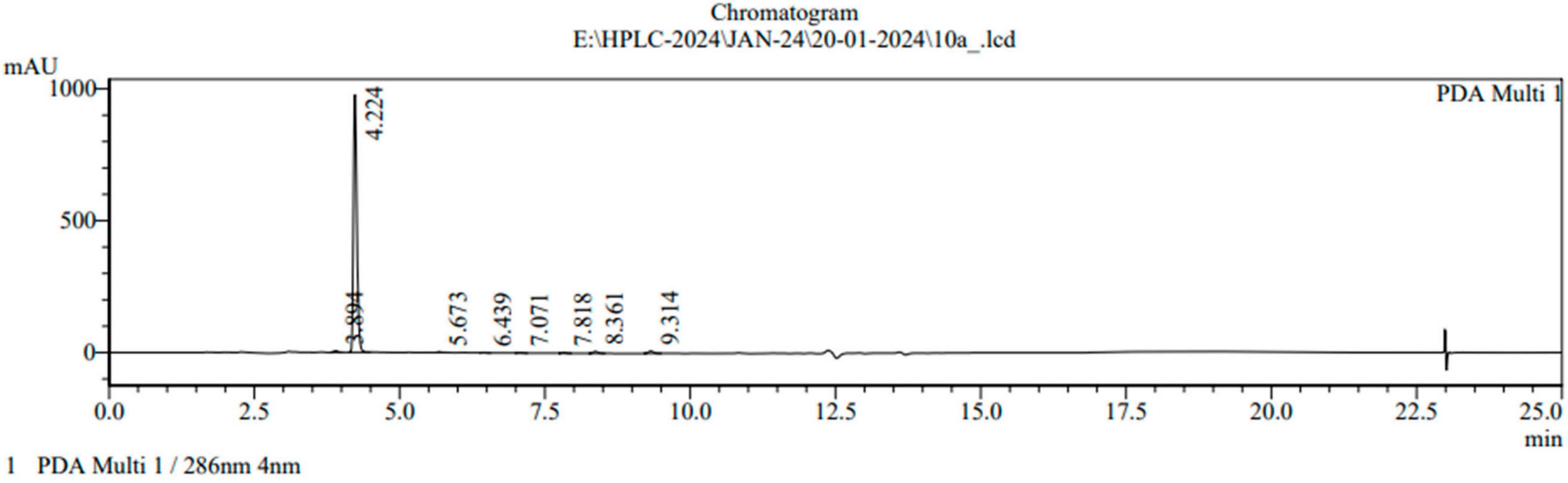

Purity= 96%

PeakTable

PDA Ch1 286nm 4nm

Peak#	Ret. Time	Area	Height	Area %
1	3.894	25743	6588	0.625
2	4.224	3960165	977634	96.135
3	5.673	12133	2625	0.295
4	6.439	6667	1415	0.162
5	7.071	7027	1376	0.171
6	7.818	12892	2744	0.313
7	8.361	41643	7337	1.011
8	9.314	53131	8856	1.290
Total		4119400	1008577	100.000

2.1. Mass spectra of compound 10b

SAMPLE INFORMATION

Sample Name:	UC-RS-08	Acquired By:	System
Sample Type:	Unknown	Date Acquired:	10-08-2024 15:26:57 IST
Vial:	1:A,6	Acq. Method Set:	ARAL_MASS_
Injection #:	1	Date Processed:	10-08-2024 15:37:35 IST, 10-08-2024
Injection Volume:	2.00 ul	Processing Methoc	Aral_Mass_
Run Time:	1.5 Minutes	Channel Name:	MS TIC
Sample Set Name	10082024_MASS	Proc. Chnl. Descr.	SQ 2: MS Scan MS TIC (2:

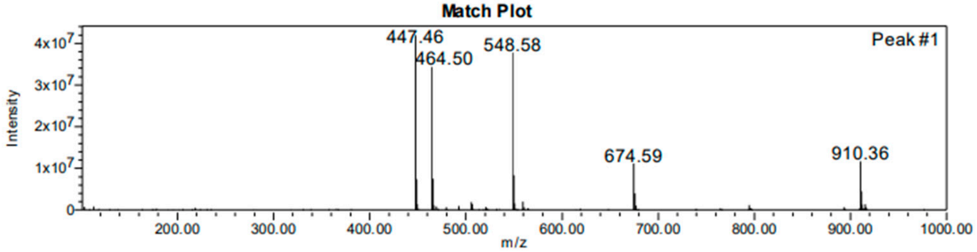

SampleName: UC-RS-08 Injection: 1 Name: Match1 Threshold: Base Peak 447.46 Channel Type 3D MS Channel Description 2: 100.00-1000.00 ES+, Centroid, CV=30

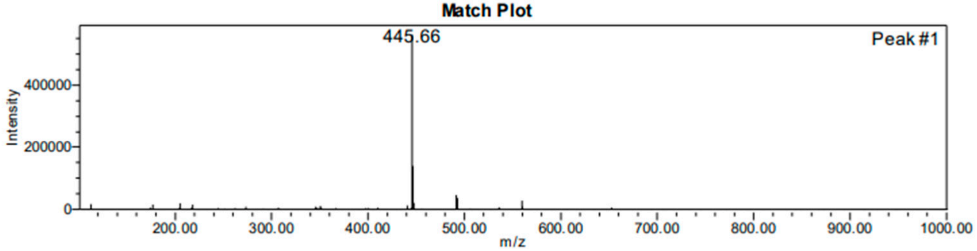

SampleName: UC-RS-08 Injection: 1 Name: Match1 Threshold: Base Peak 445.66 Channel Type 3D MS Channel Description 5: 100.00-1000.00 ES-, Centroid, CV=50

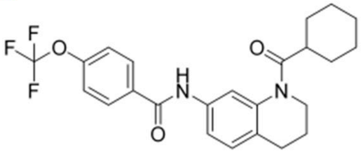

Exact Mass: 446.18
Molecular Weight: 446.47

2.2. ^{1}H NMR of compound 10b

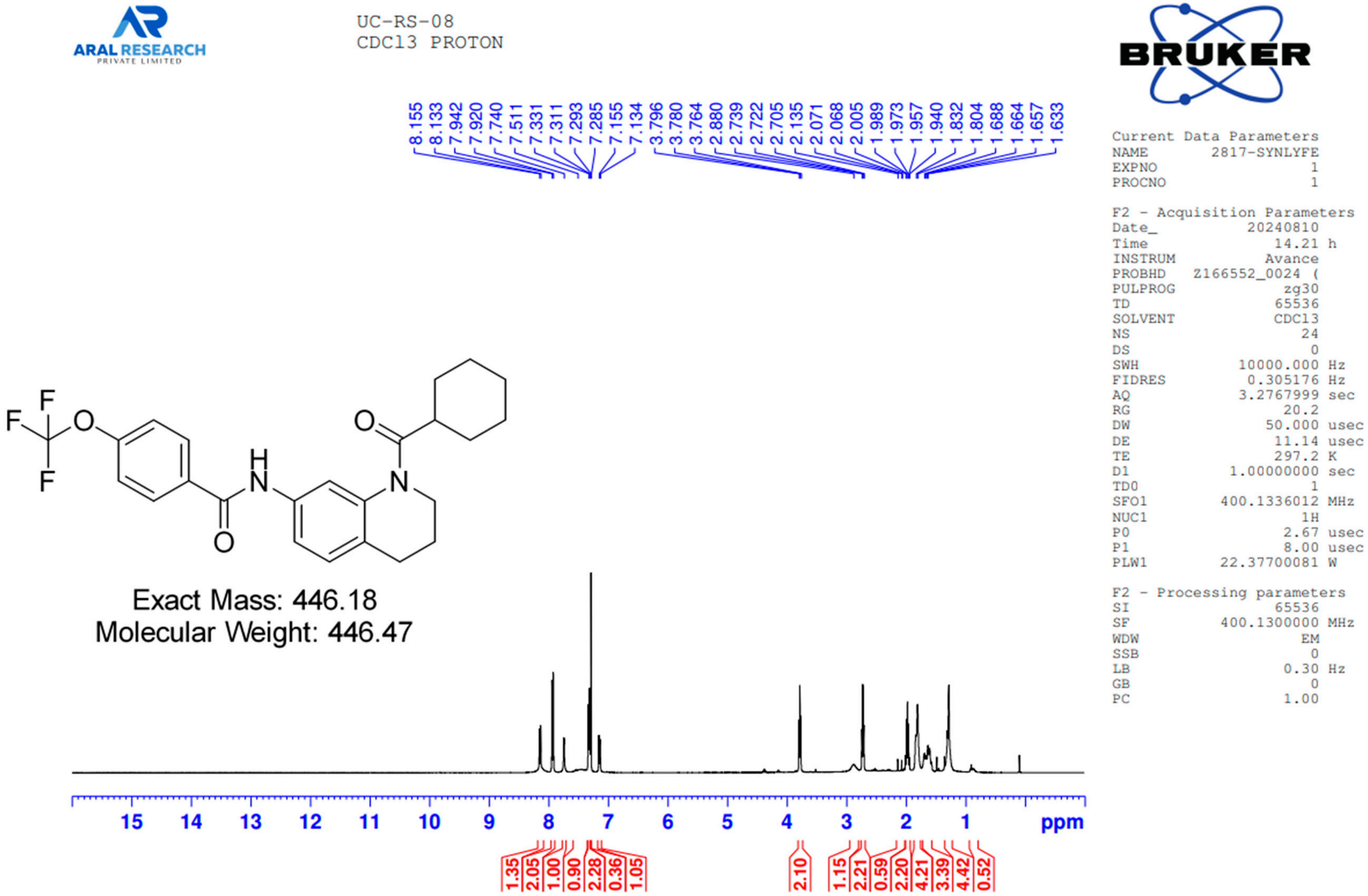

2.3. ^{13}C NMR of compound 10b

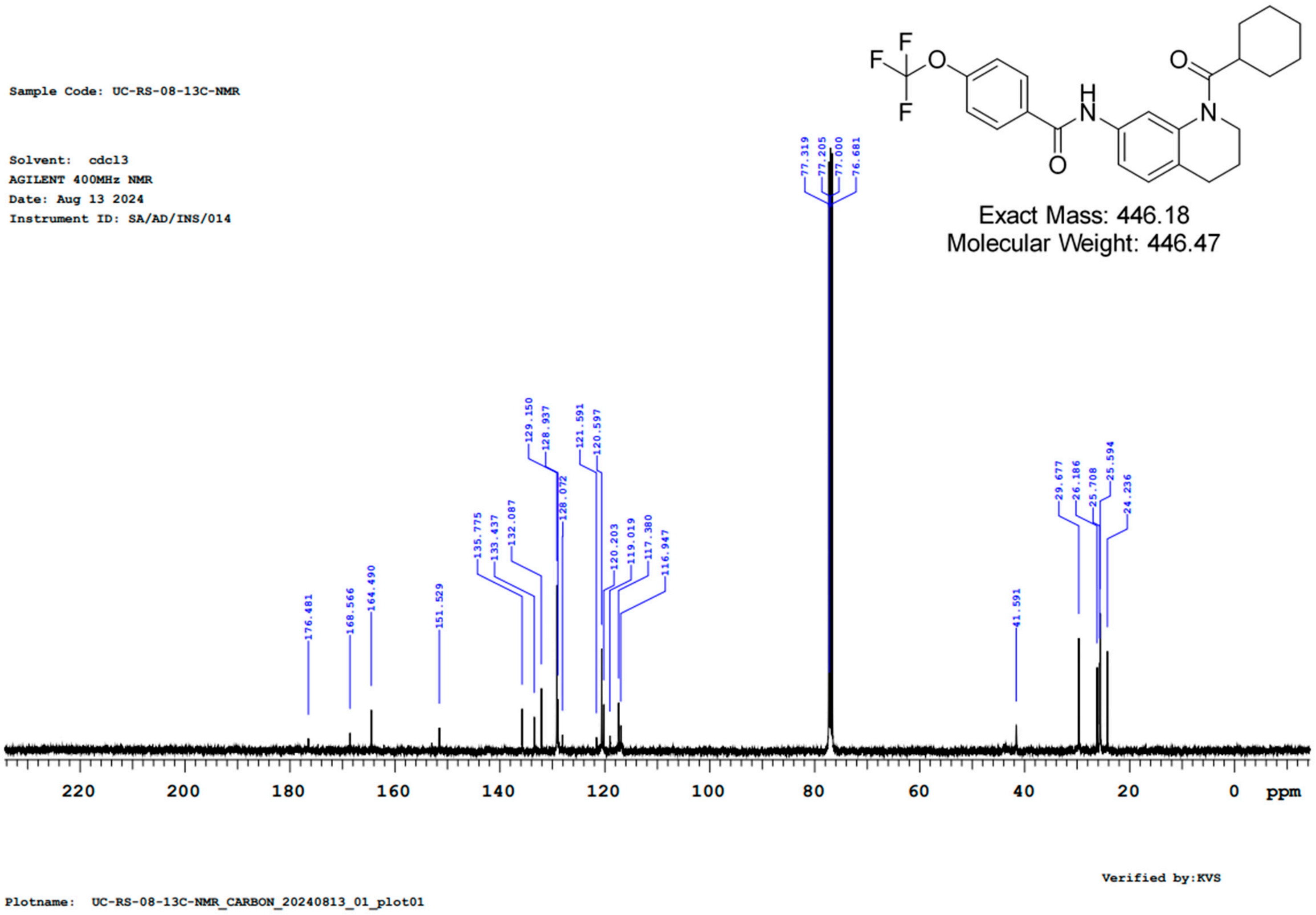

2.4. HPLC chromatogram of compound 10b

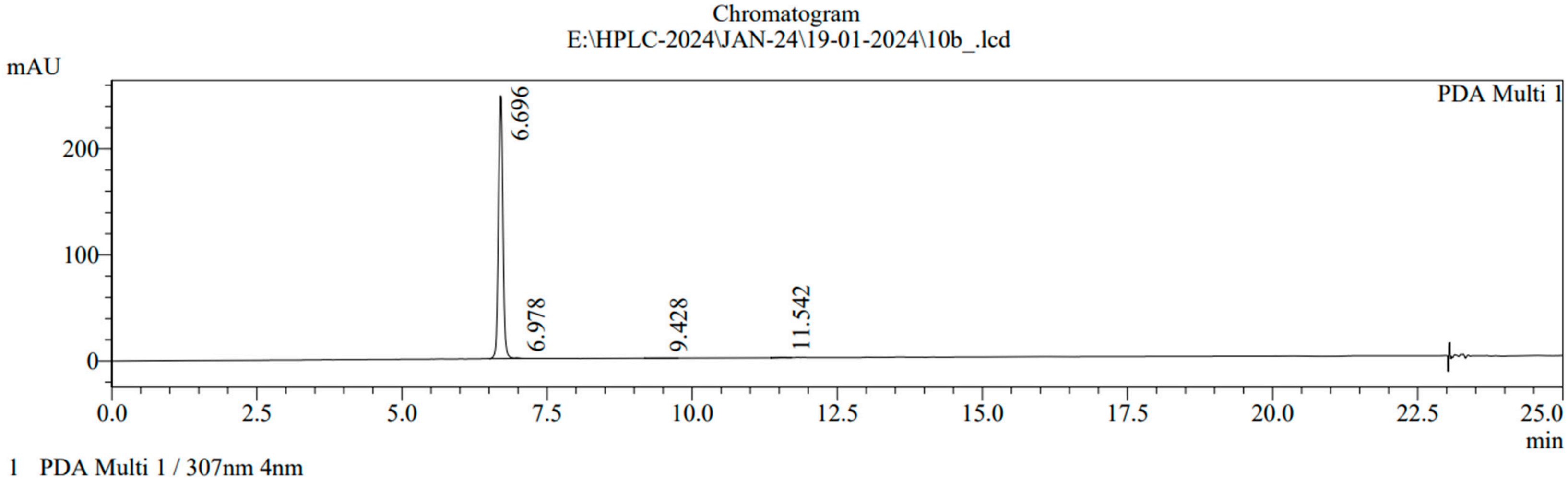

PeakTable

PDA Ch1 307nm 4nm

Peak#	Ret. Time	Area	Height	Area %
1	6.696	1343244	247726	99.066
2	6.978	2576	543	0.190
3	9.428	7844	450	0.579
4	11.542	2243	216	0.165
Total		1355907	248934	100.000

Purity= 99%

3.1. Mass spectra of compound 10c

SAMPLE INFORMATION

Sample Name:	UE-RS-12	Acquired By:	System
Sample Type:	Unknown	Date Acquired:	09-12-2024 09:15:29 IST
Vial:	1:C,4	Acq. Method Set:	ARAL_MASS
Injection #:	1	Date Processed:	09-12-2024 09:19:28 IST, 09-12-2024
Injection Volume:	2.00 ul	Processing Methoc	Aral_Mass_
Run Time:	1.5 Minutes	Channel Name:	MS TIC
Sample Set Name	09122024_MASS	Proc. Chnl. Descr.	SQ 2: MS Scan MS TIC (2: [illegible]

Match Plot

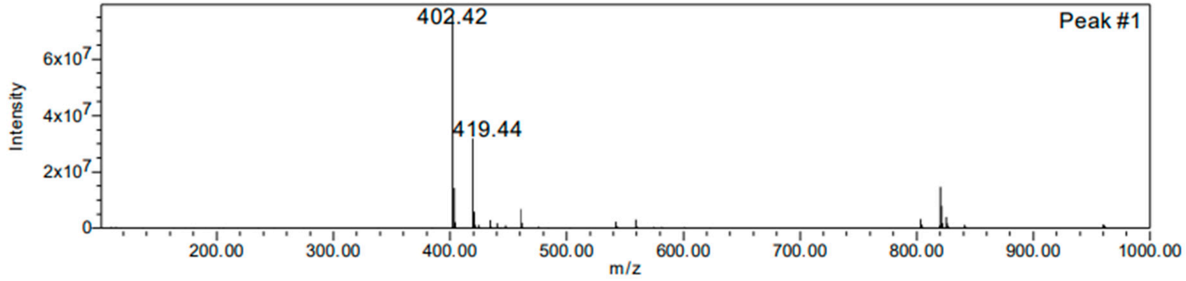

SampleName: UE-RS-12 Injection: 1 Name: Match1 Threshold: Base Peak 402.42 Channel Type 3D MS Channel Description 2: 100.00-1000.00 ES+, Centroid, CV=30

Match Plot

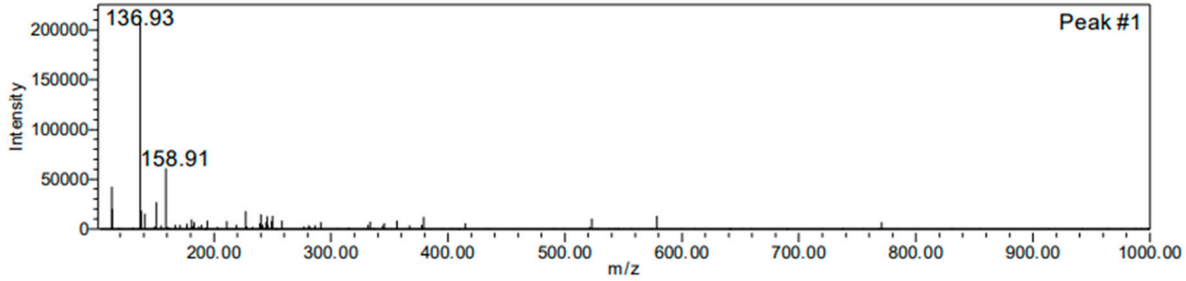

SampleName: UE-RS-12 Injection: 1 Name: Match1 Threshold: Base Peak 136.93 Channel Type 3D MS Channel Description 5: 100.00-1000.00 ES-, Centroid, CV=50

3.2. ¹H NMR of compound 10c

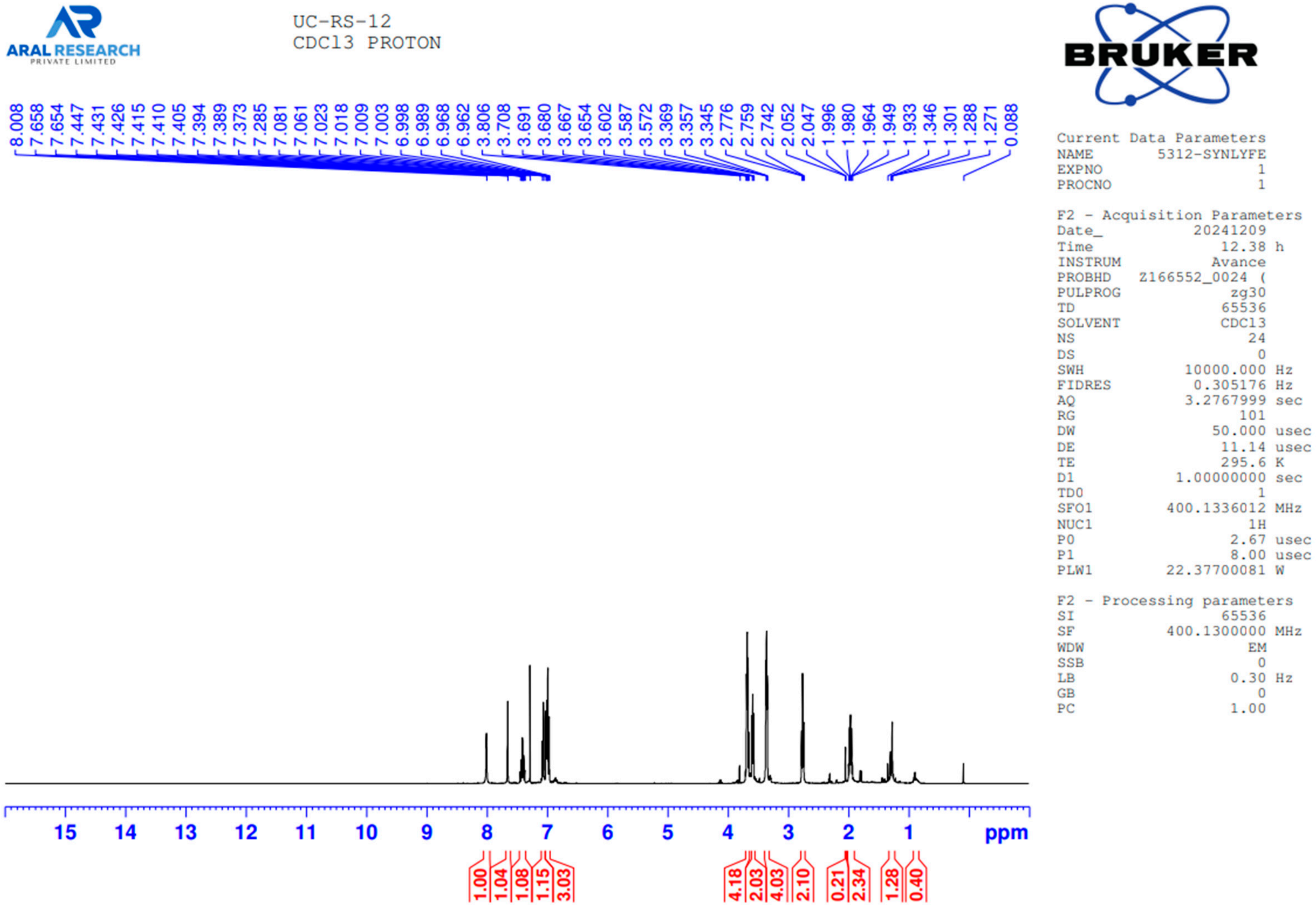

3.3. ¹³C NMR of compound 10c

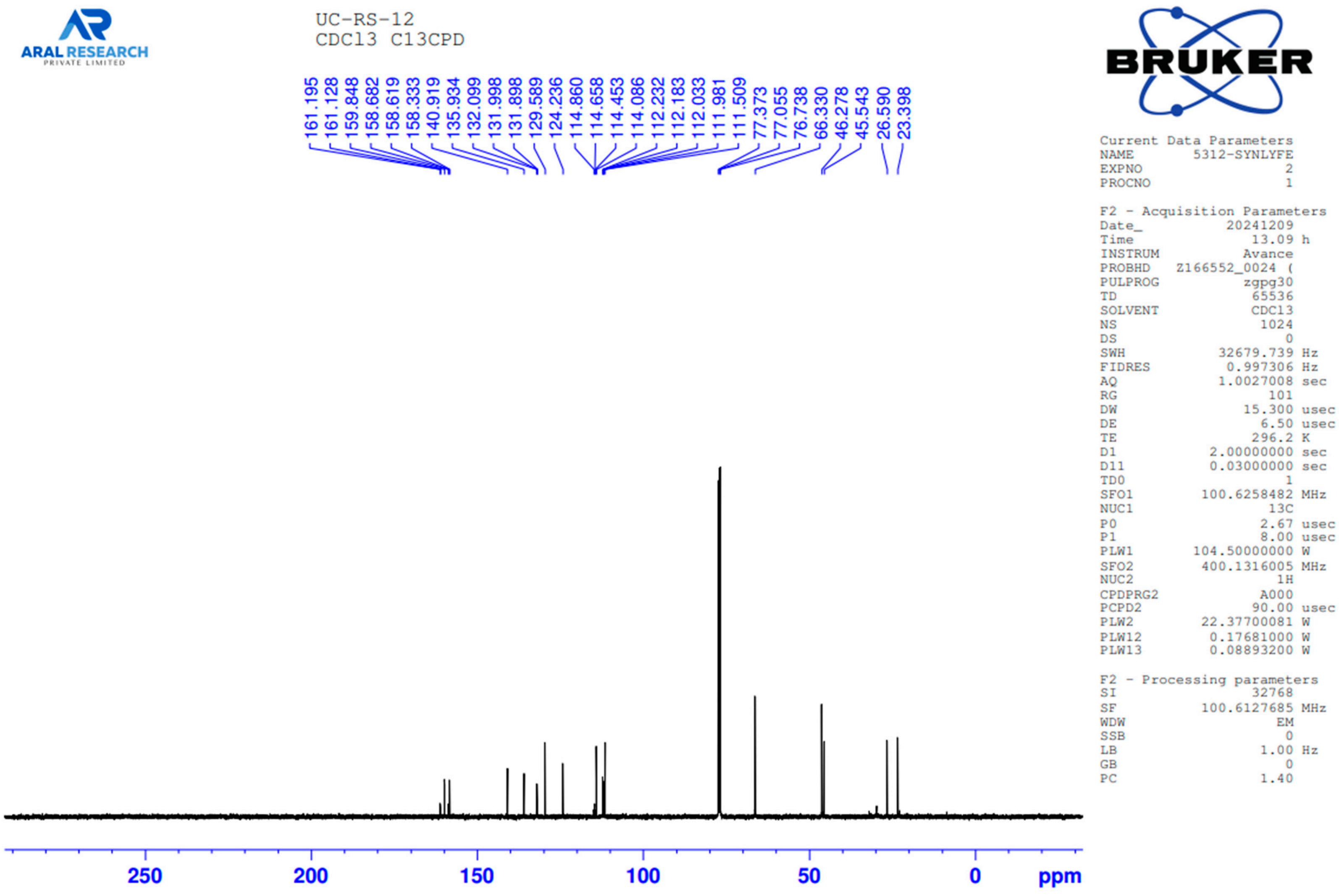

3.4. HPLC chromatogram of compound 10c

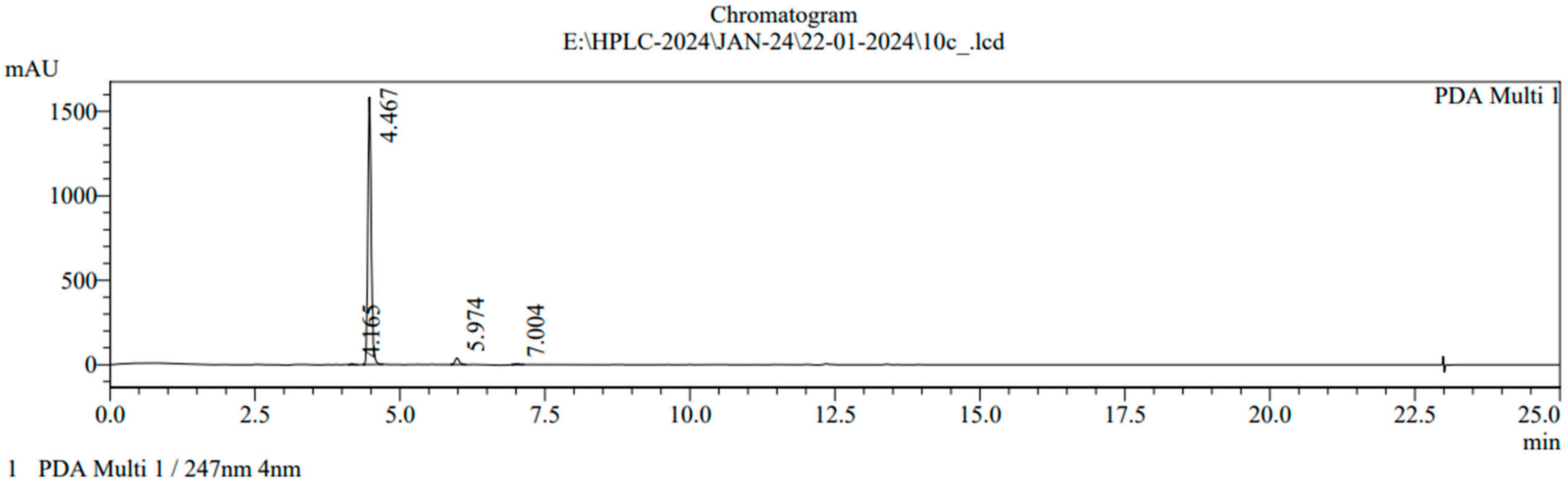

PeakTable

PDA Ch1 247nm 4nm

Peak#	Ret. Time	Area	Height	Area %
1	4.165	15363	4216	0.239
2	4.467	6196651	1583187	96.356
3	5.974	192055	38473	2.986
4	7.004	26912	5363	0.418
Total		6430981	1631238	100.000

Purity: 96%

4.1. Mass spectra of compound 10d

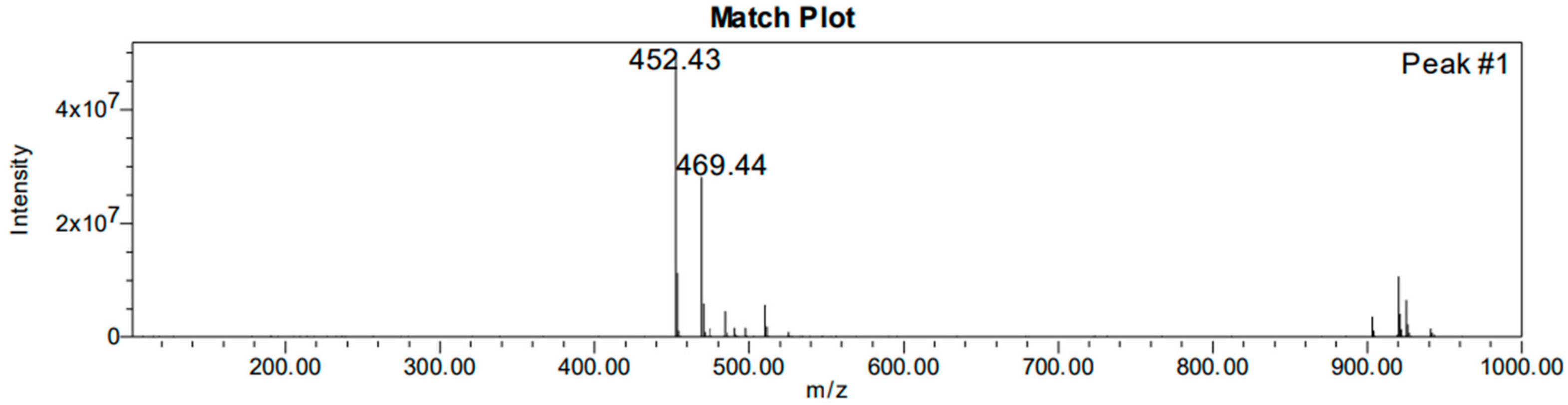

SampleName: UE-RS-13 Injection: 1 Name: Match1 Threshold: Base Peak 452.43 Channel Type 3D MS Channel Description 2: 100.00-1000.00 ES+, Centroid, CV=30

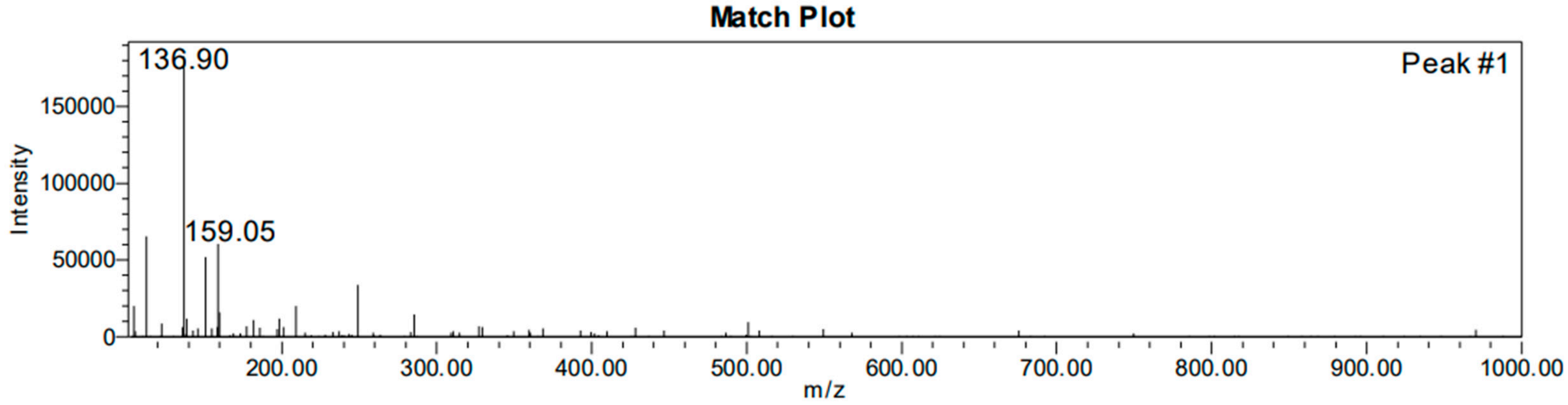

SampleName: UE-RS-13 Injection: 1 Name: Match1 Threshold: Base Peak 136.90 Channel Type 3D MS Channel Description 5: 100.00-1000.00 ES-, Centroid, CV=50

4.2. ^{1}H NMR of compound 10d

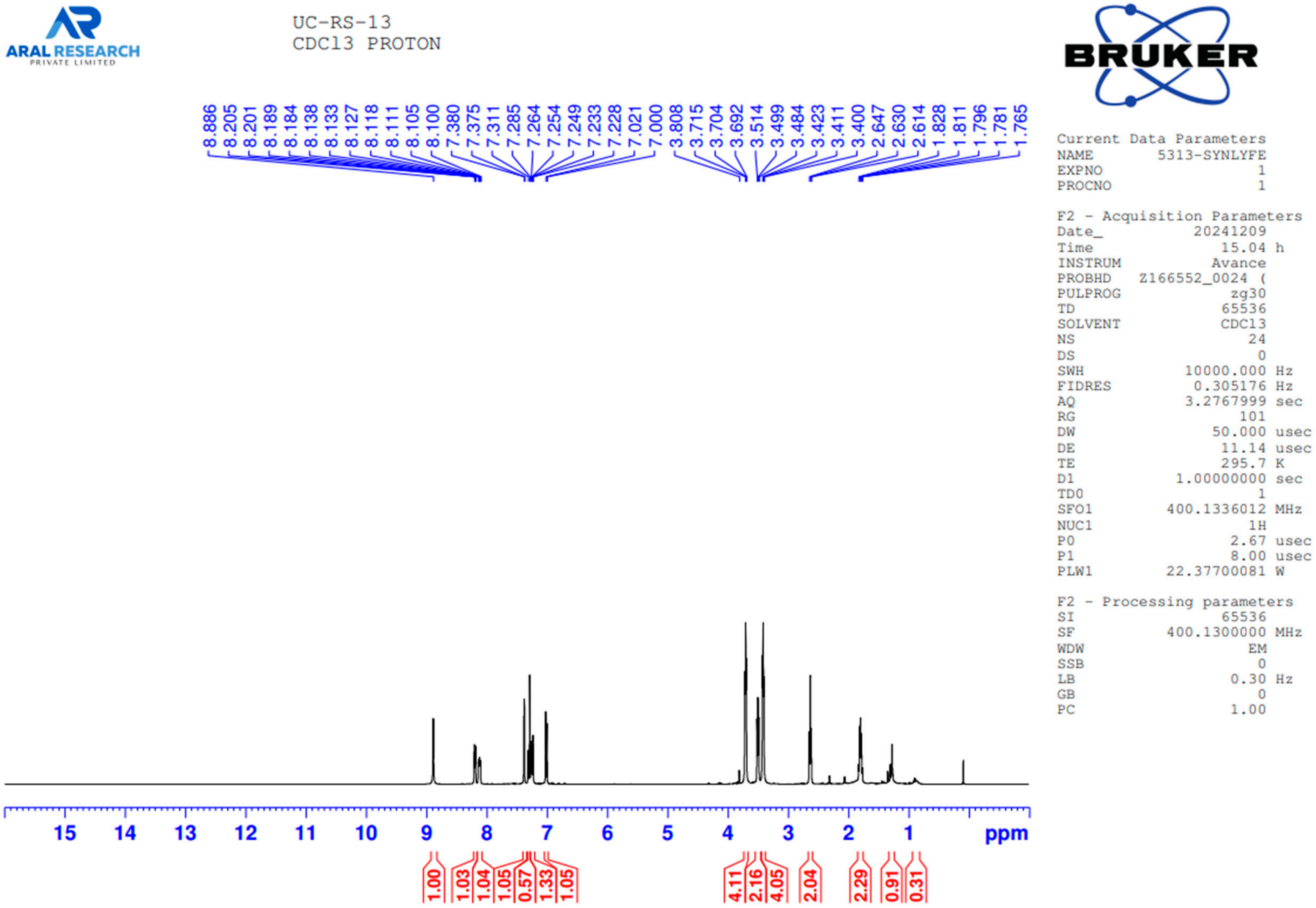

4.3. ^{13}C NMR of compound 10d

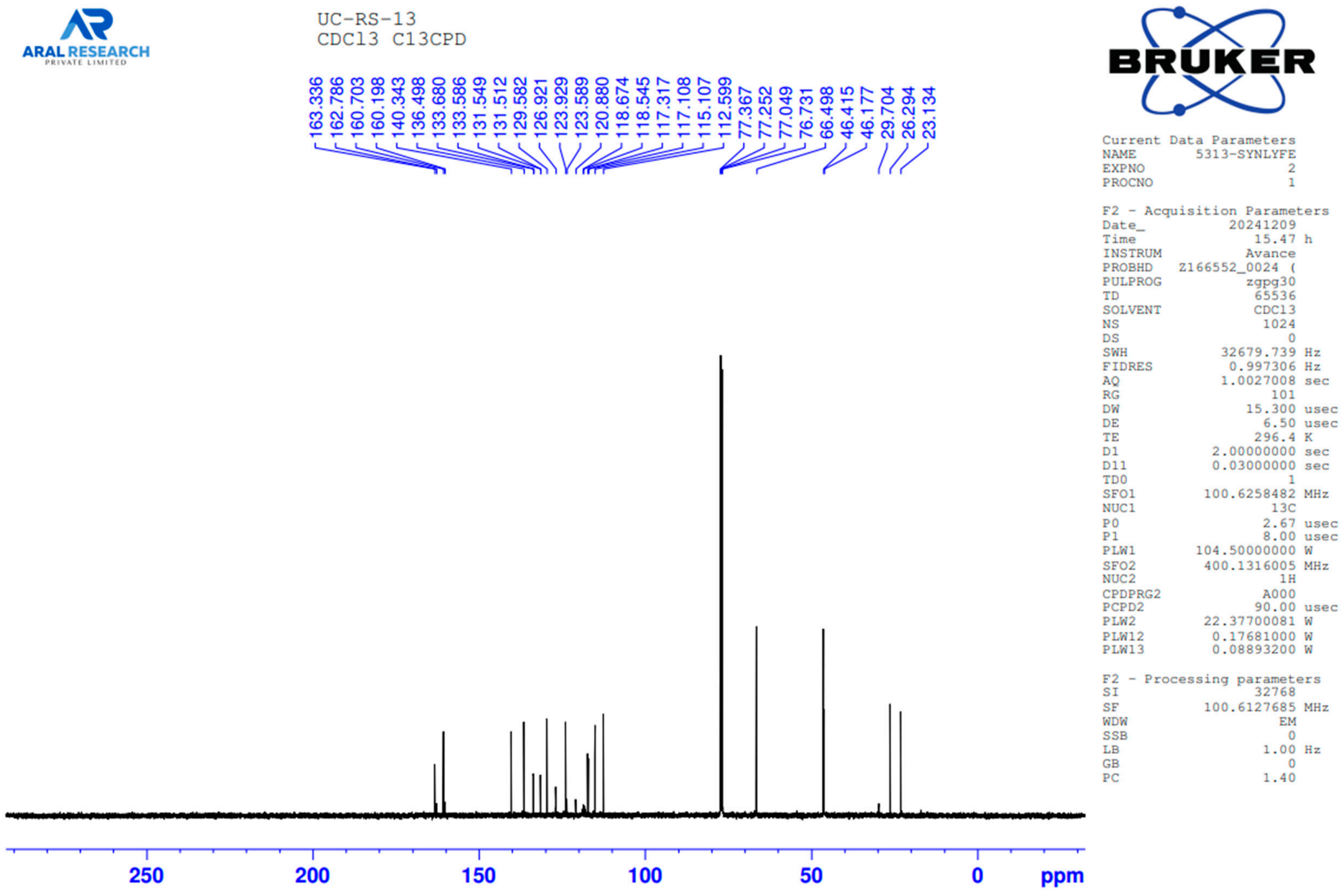

4.4. HPLC chromatogram of compound 10d

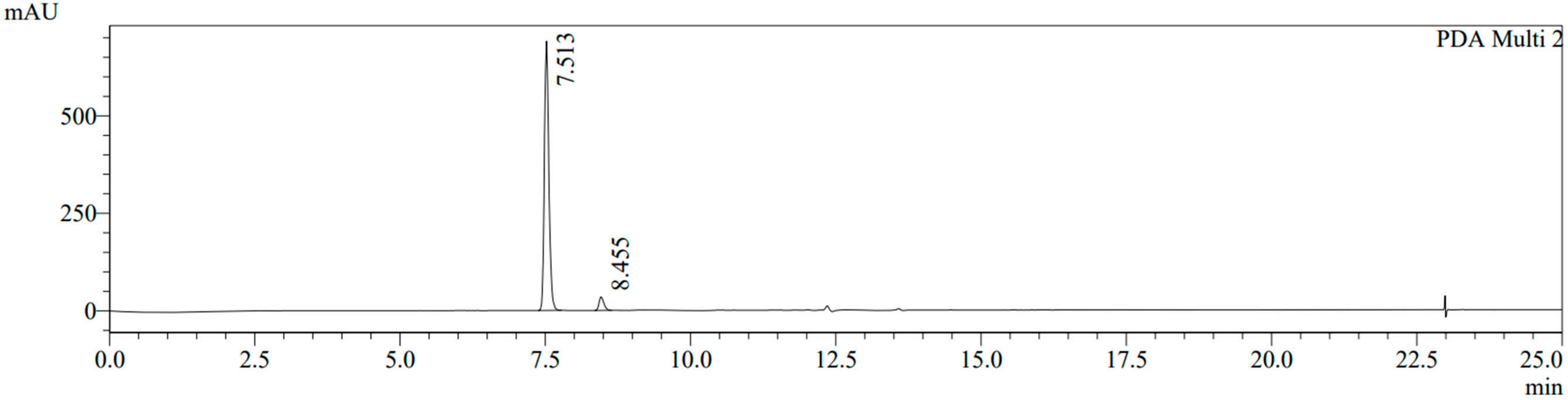

1 PDA Multi 1 / 242nm 4nm
2 PDA Multi 2 / 285nm 4nm

Purity= 99%

PeakTable

PDA Ch1 242nm 4nm

Peak#	Ret. Time	Area	Height	Area %
1	7.513	12435022	2308250	99.622
2	8.456	47148	8713	0.378
Total		12482170	2316963	100.000

5.1. Mass spectra of compound 10e

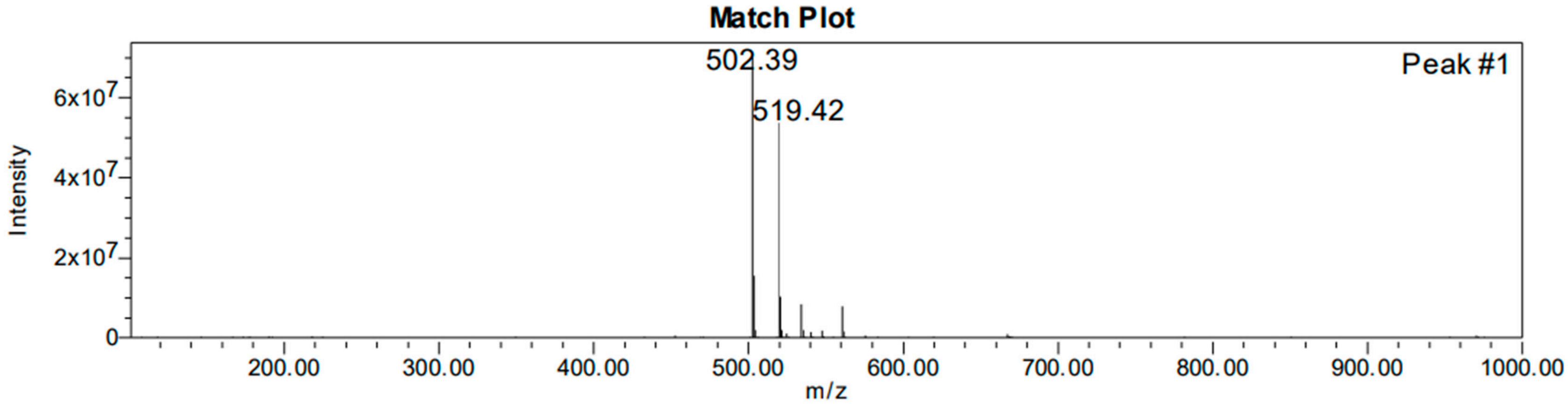

SampleName: UE-RS-14 Injection: 1 Name: Match1 Threshold: Base Peak 502.39 Channel Type 3D MS Channel Description 2: 100.00-1000.00 ES+, Centroid, CV=30

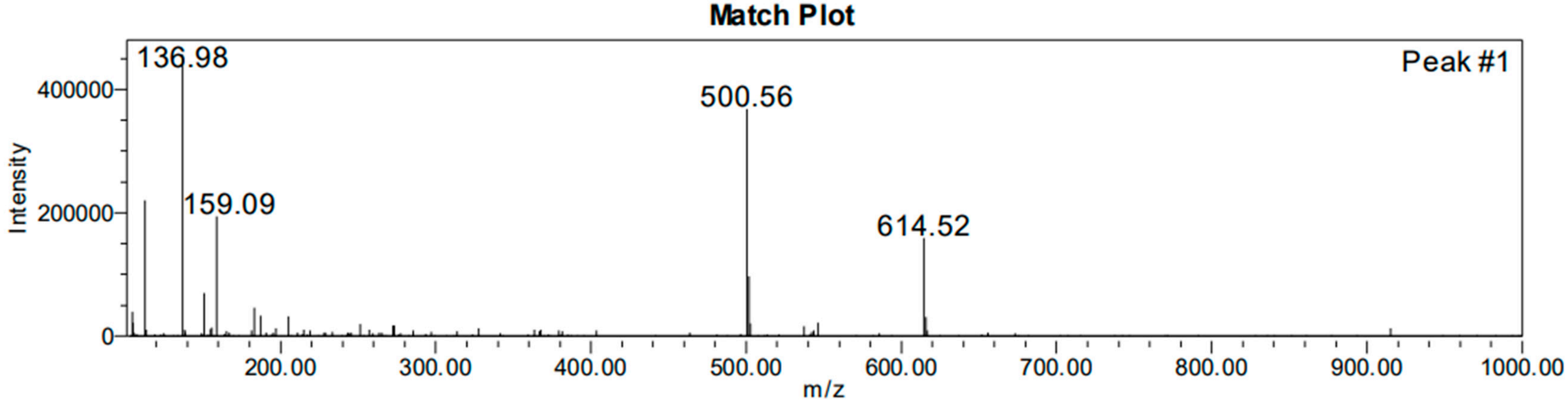

SampleName: UE-RS-14 Injection: 1 Name: Match1 Threshold: Base Peak 136.98 Channel Type 3D MS Channel Description 5: 100.00-1000.00 ES-, Centroid, CV=50

5.2. ^{1}H NMR of compound 10e

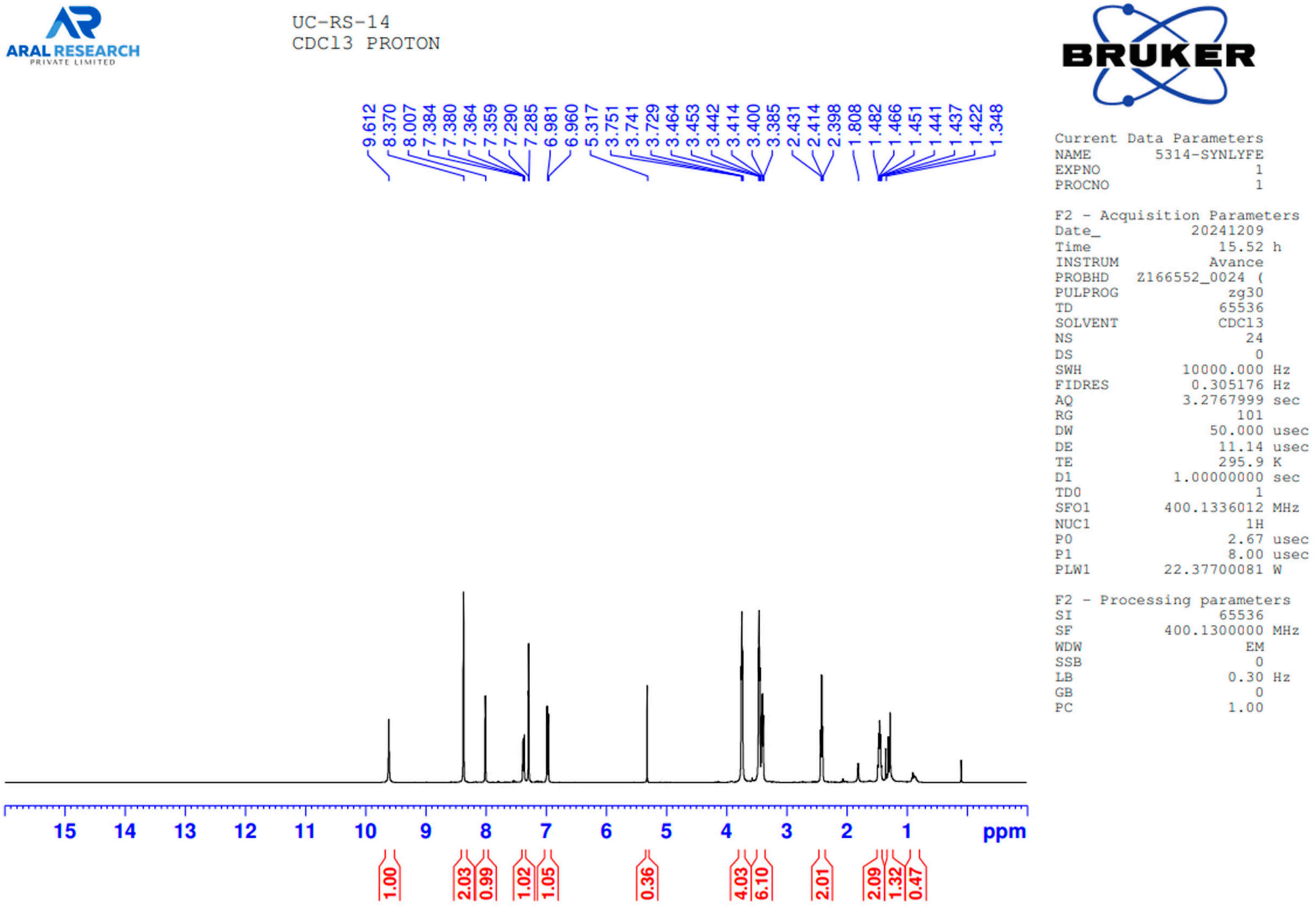

5.3. ^{13}C NMR of compound 10e

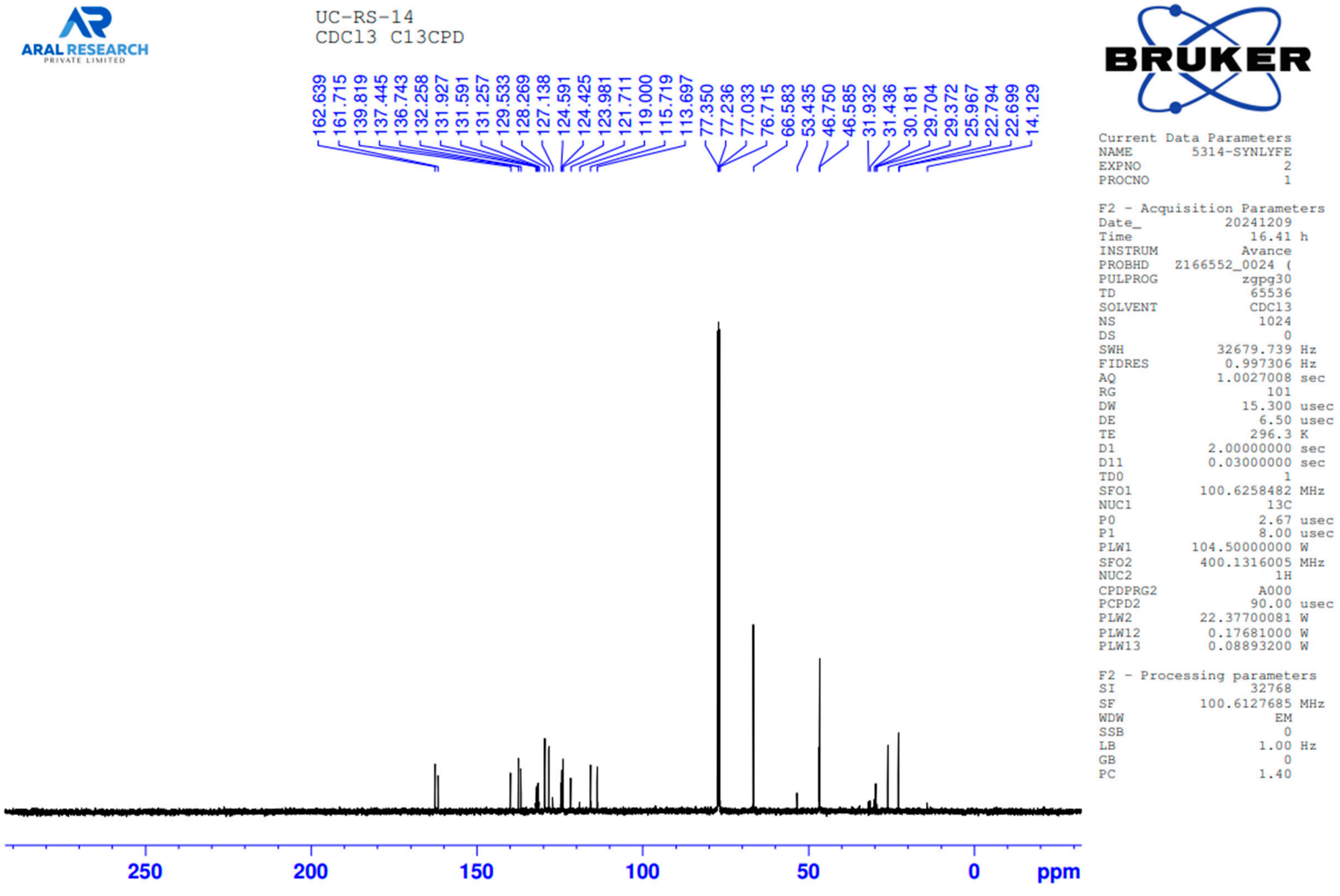

5.4. HPLC chromatogram of compound 10e

Sample Name: 10e

Acquired by	: Admin
Date Acquired	: 1/17/2024 12:20:41 PM
Sample ID	: O2Hr
Vial#	: 61
Injection Volume	: 10
Data File	: 10e_-.lcd
Method file	:250_SUNFIRE_NORMAL METHOD.lcm

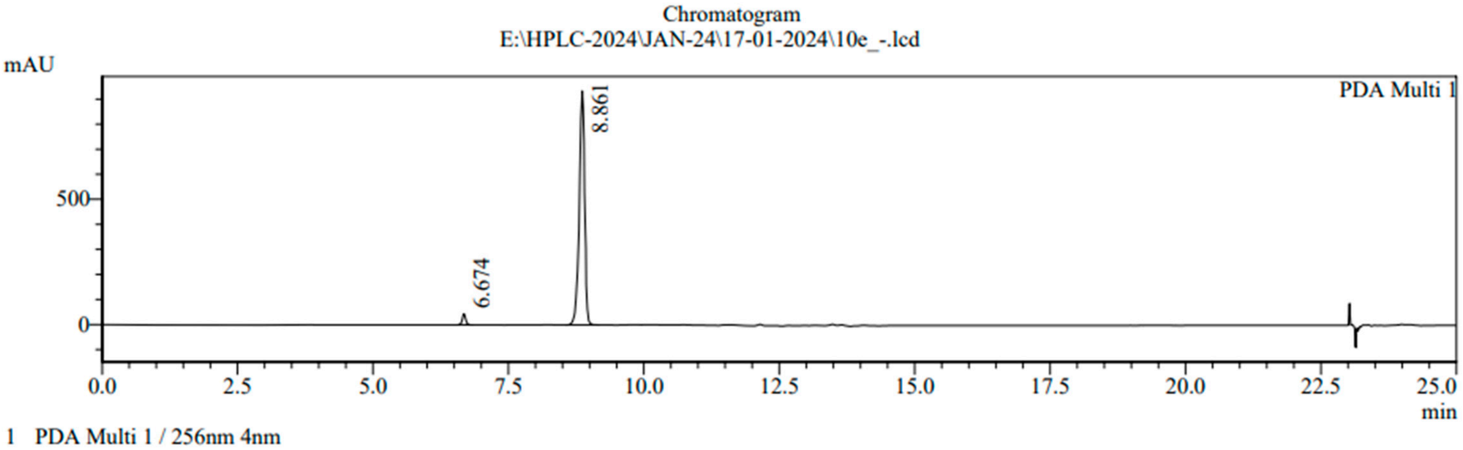

PeakTable

PDA Ch1 256nm 4nm

Peak#	Ret. Time	Area	Height	Area %
1	6.674	191372	43806	2.871
2	8.861	6473598	934607	97.129
Total		6664971	978413	100.000

Purity: 97%

6.1. Mass spectra of compound 10f

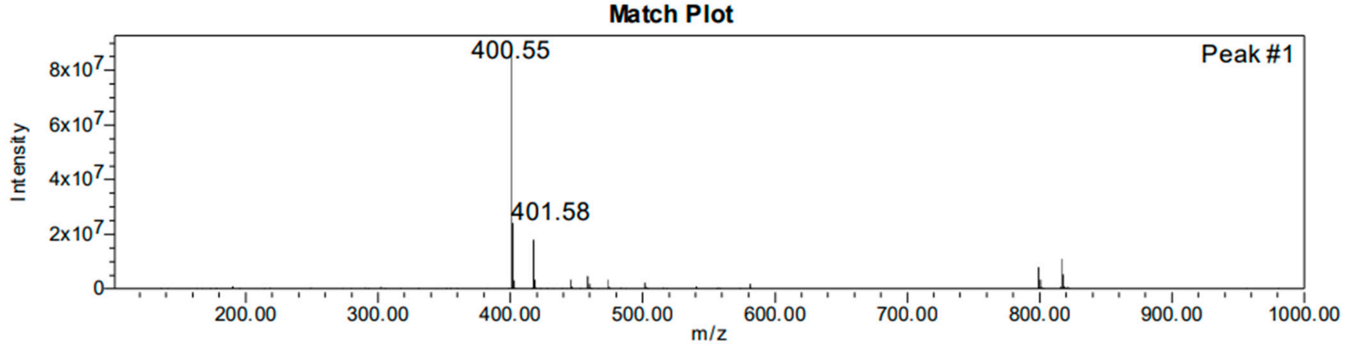

SampleName: UC-RS-09 Injection: 1 Name: Match1 Threshold: Base Peak 400.55 Channel Type 3D MS Channel Description 2: 100.00-1000.00 ES+, Centroid, CV=30

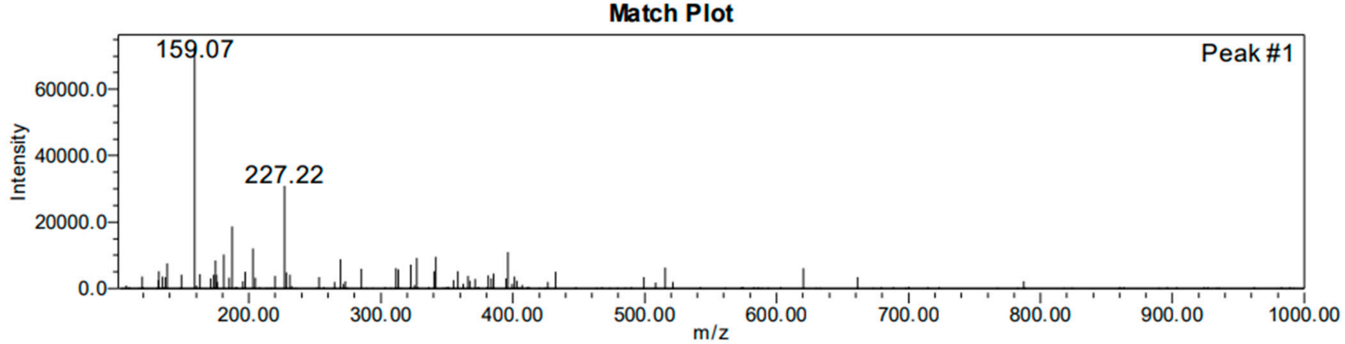

SampleName: UC-RS-09 Injection: 1 Name: Match1 Threshold: Base Peak 159.07 Channel Type 3D MS Channel Description 5: 100.00-1000.00 ES-, Centroid, CV=50

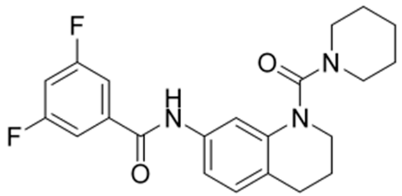

Exact Mass: 399.18
Molecular Weight: 399.44

6.2. ^{1}H NMR of compound 10f

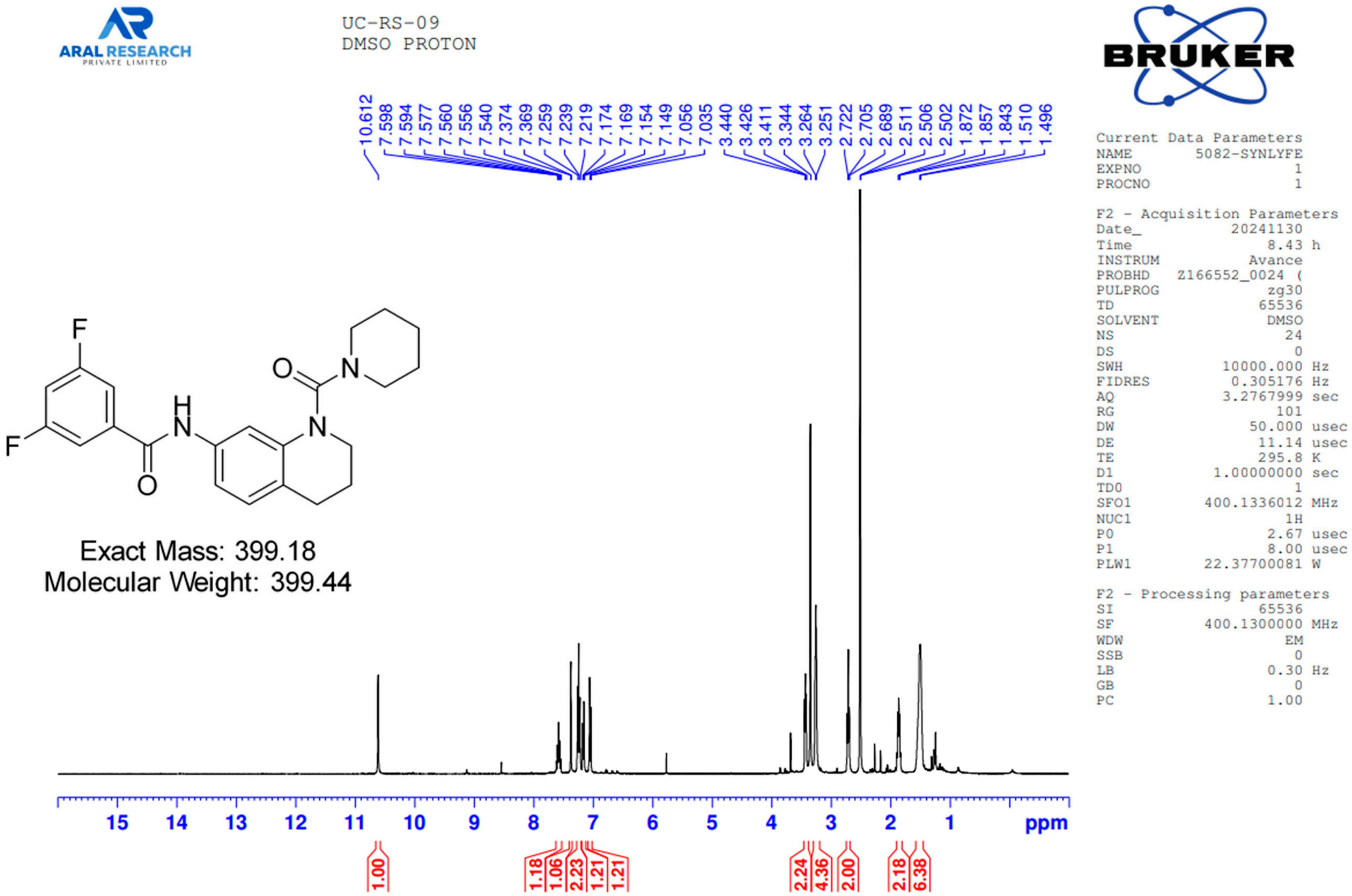

6.3. ^{13}C NMR of compound 10f

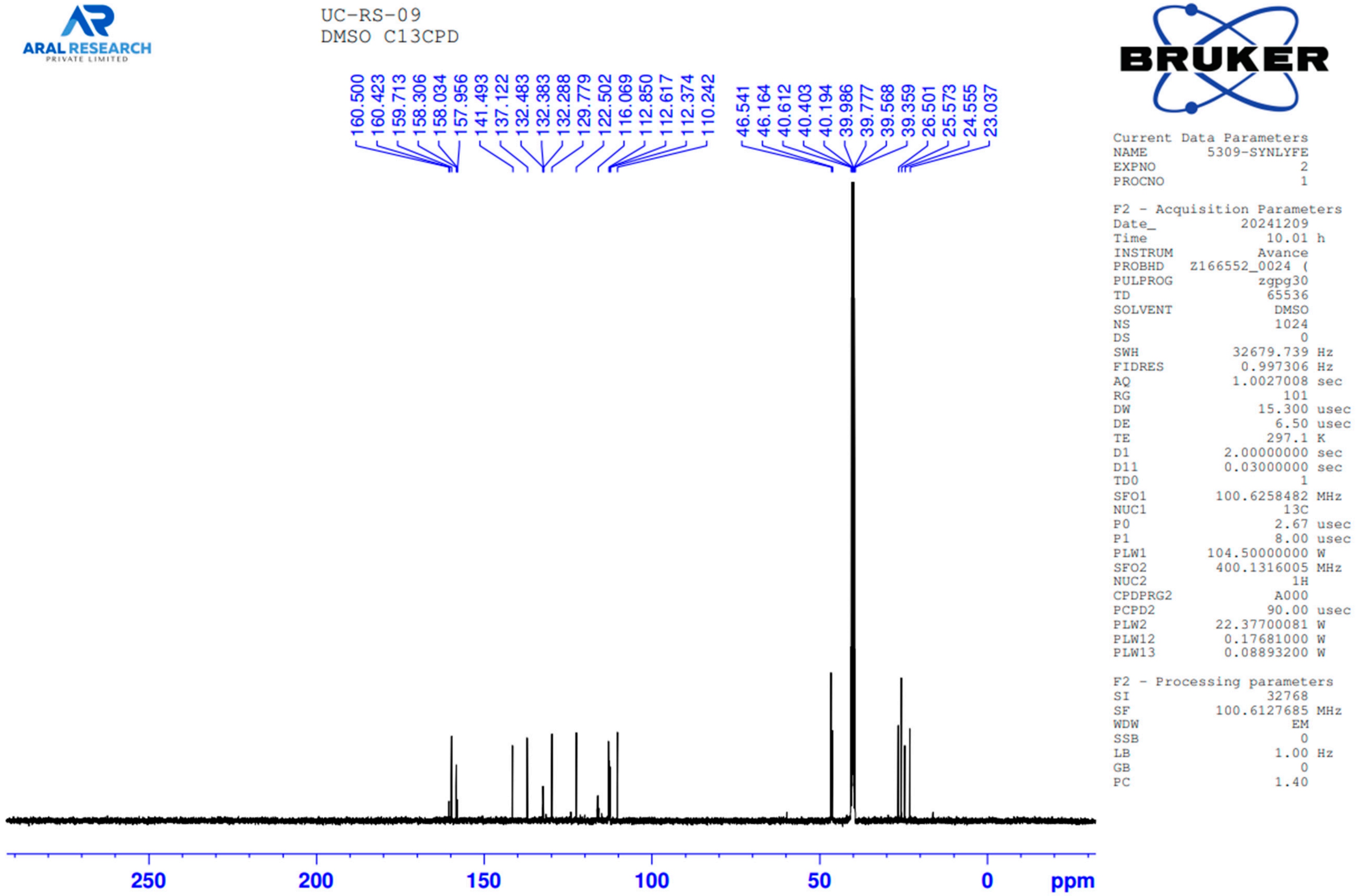

6.4. HPLC chromatogram of compound 10f

Sample Name: 10f

Acquired by	: Admin
Date Acquired	: 1/21/2024 8:01:33 PM
Sample ID	: O2Hr
Vial#	: 24
Injection Volume	: 40
Data File	: 10f---.lcd
Method file	:250_SUNFIRE_NORMAL METHOD.lcm

Chromatogram
E:\HPLC-2024\JAN-24\21-01-2024\10f---.lcd

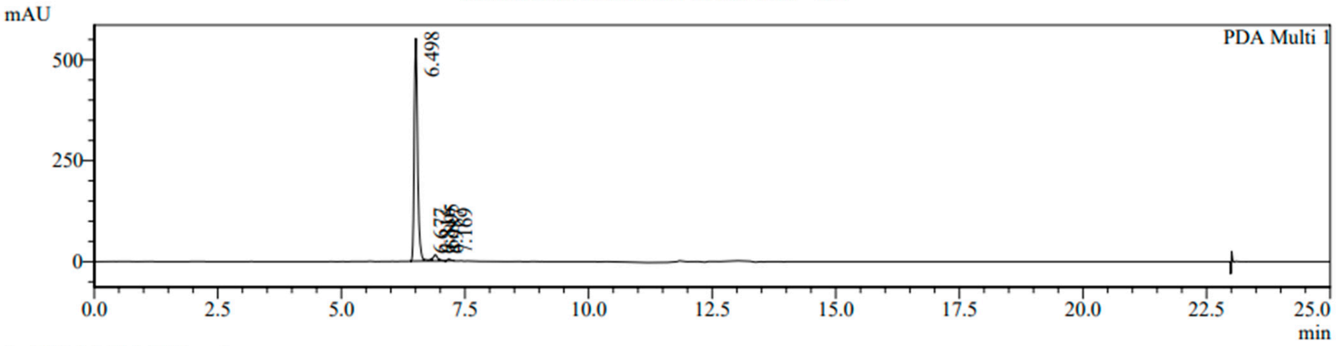

1 PDA Multi 1 / 254nm 4nm

PeakTable

PDA Ch1 254nm 4nm

Peak#	Ret. Time	Area	Height	Area %
1	6.498	2624011	551968	94.570
2	6.677	17573	3755	0.633
3	6.816	16016	5227	0.577
4	6.895	87852	15335	3.166
5	6.987	9848	3355	0.355
6	7.169	19364	4367	0.698
Total		2774664	584007	100.000

Purity: 95%

7.1. Mass spectra of compound 10g

S A M P L E   I N F O R M A T I O N

Sample Name:	UC-RS-10	Acquired By:	System
Sample Type:	Unknown	Date Acquired:	30-11-2024 09:00:11 IST
Vial:	1:A,4	Acq. Method Set:	ARAL_MASS
Injection #:	1	Date Processed:	30-11-2024 09:12:49 IST, 30-11-2024
Injection Volume:	2.00 ul	Processing Methoc	Aral_Mass_
Run Time:	1.5 Minutes	Channel Name:	MS TIC
Sample Set Name	30112024_MASS	Proc. Chnl. Descr.	SQ 2: MS Scan MS TIC (2:

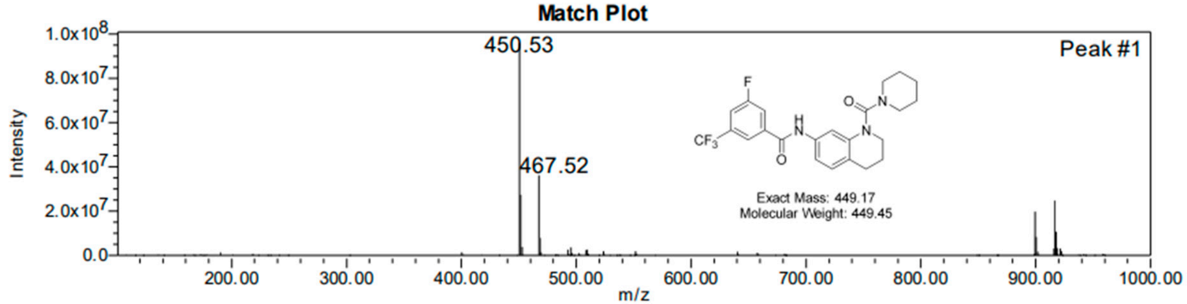

SampleName: UC-RS-10 Injection: 1 Name: Match1 Threshold: Base Peak 450.53 Channel Type 3D MS Channel Description 2: 100.00-1000.00 ES+, Centroid, CV=30

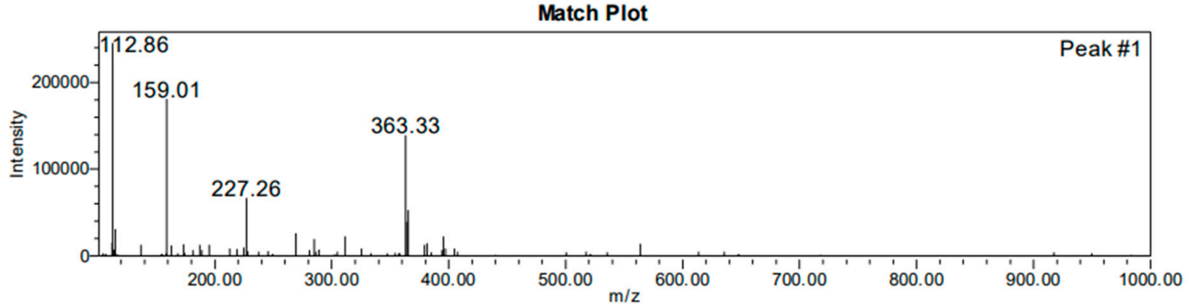

SampleName: UC-RS-10 Injection: 1 Name: Match1 Threshold: Base Peak 112.86 Channel Type 3D MS Channel Description 5: 100.00-1000.00 ES-, Centroid, CV=50

7.2. ^{1}H NMR of compound 10g

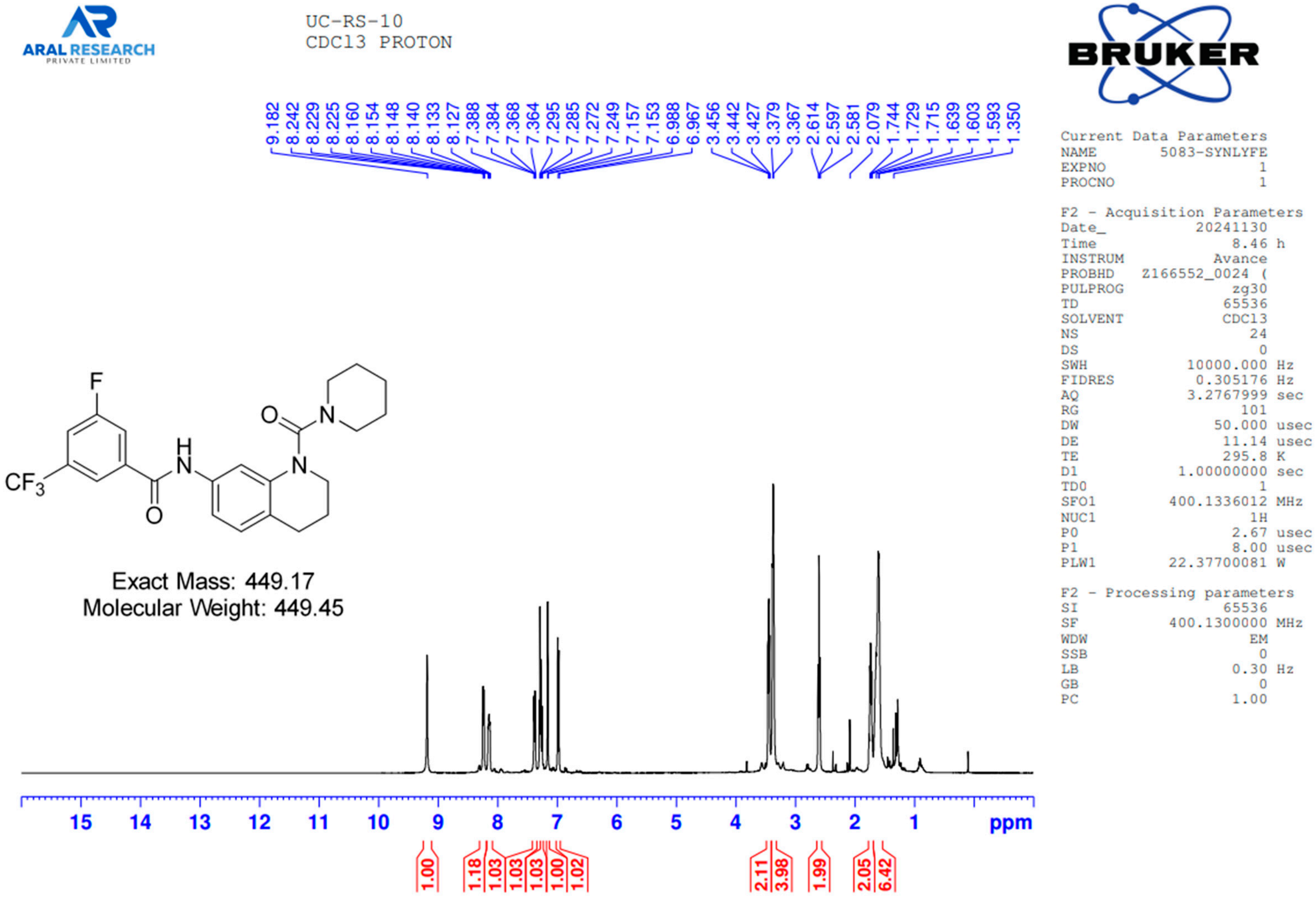

7.3. ^{13}C NMR of compound 10g

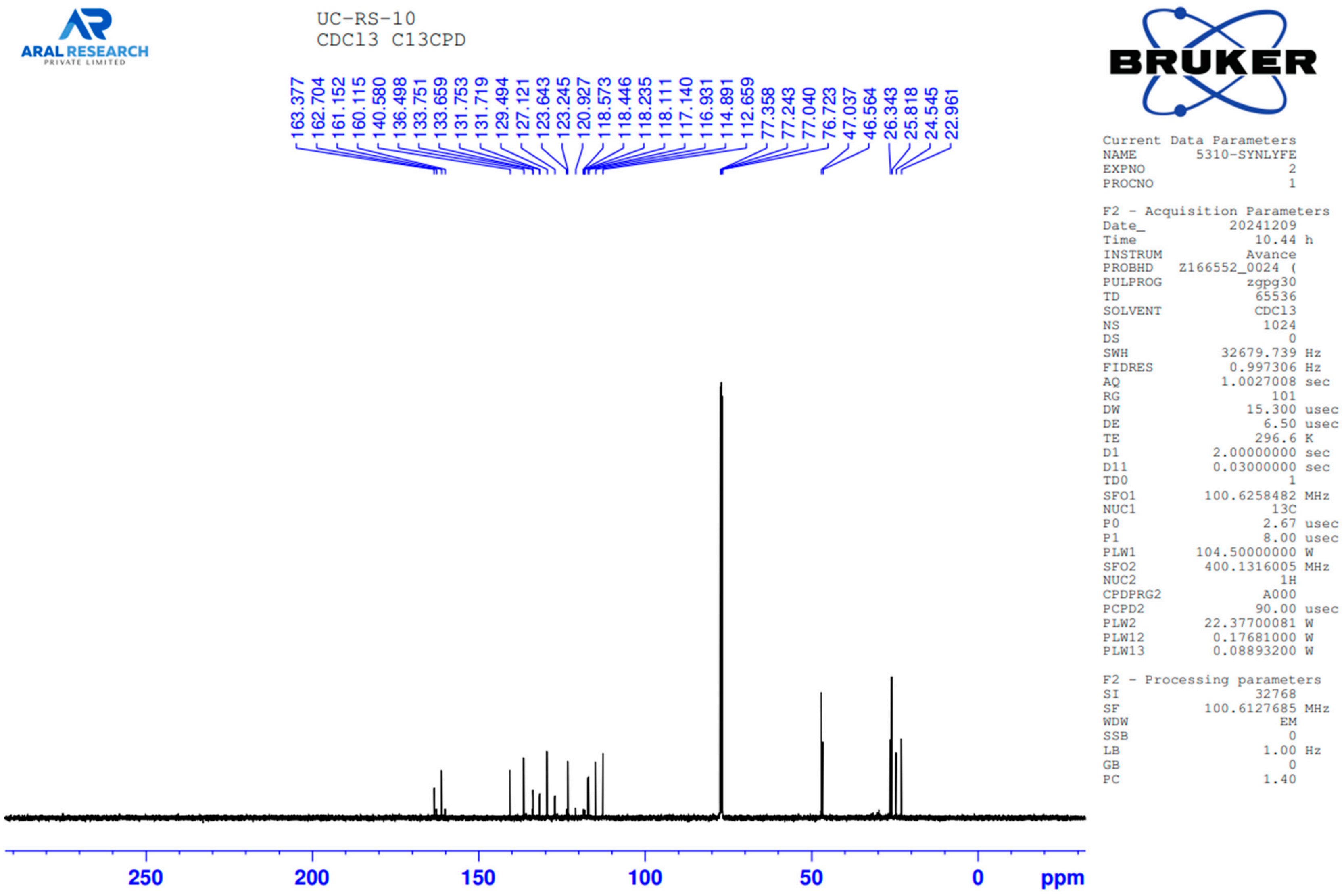

7.4. HPLC chromatogram of compound 10g

Sample Name: 10g

Acquired by	: Admin
Date Acquired	: 1/19/2024 11:49:26 AM
Sample ID	: 4
Vial#	: 4
Injection Volume	: 15
Data File	: 10g__.lcd
Method file	:250_SUNFIRE_NORMAL METHOD.lcm

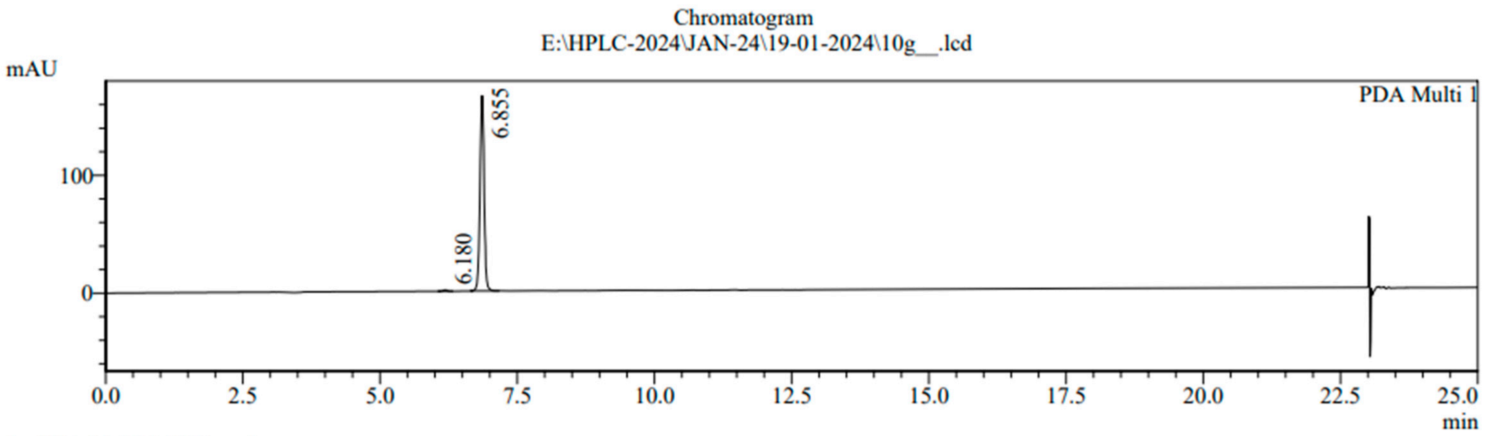

1 PDA Multi 1 / 299nm 4nm

Purity: 99%

PeakTable

PDA Ch1 299nm 4nm

Peak#	Ret. Time	Area	Height	Area %
1	6.180	5666	1153	0.638
2	6.855	882352	165799	99.362
Total		888018	166952	100.000

8.1. Mass spectra of compound 10h

SAMPLE INFORMATION

Sample Name:	UC-RS-11	Acquired By:	System
Sample Type:	Unknown	Date Acquired:	30-11-2024 09:02:38 IST
Vial:	1:A,5	Acq. Method Set:	ARAL_MASS
Injection #:	1	Date Processed:	30-11-2024 09:15:08 IST, 30-11-2024
Injection Volume:	2.00 ul	Processing Methoc	Aral_Mass_
Run Time:	1.5 Minutes	Channel Name:	MS TIC
Sample Set Name	30112024_MASS	Proc. Chnl. Descr.	SQ 2: MS Scan MS TIC (2:

Match Plot

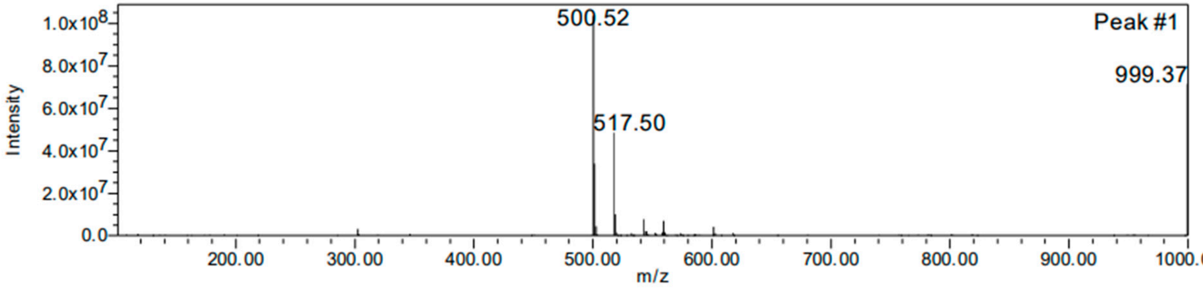

SampleName: UC-RS-11 Injection: 1 Name: Match1 Threshold: Base Peak 500.52 Channel Type 3D MS Channel Description 2: 100.00-1000.00 ES+, Centroid, CV=30

Match Plot

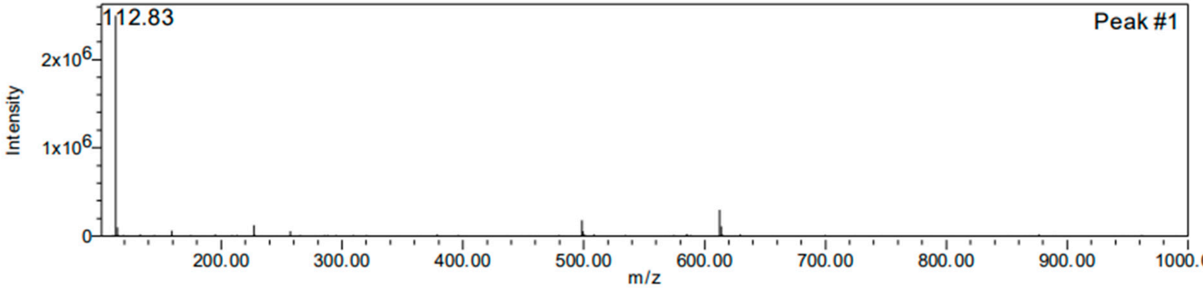

SampleName: UC-RS-11 Injection: 1 Name: Match1 Threshold: Base Peak 112.83 Channel Type 3D MS Channel Description 5: 100.00-1000.00 ES-, Centroid, CV=50

8.2. ^{1}H NMR of compound 10h

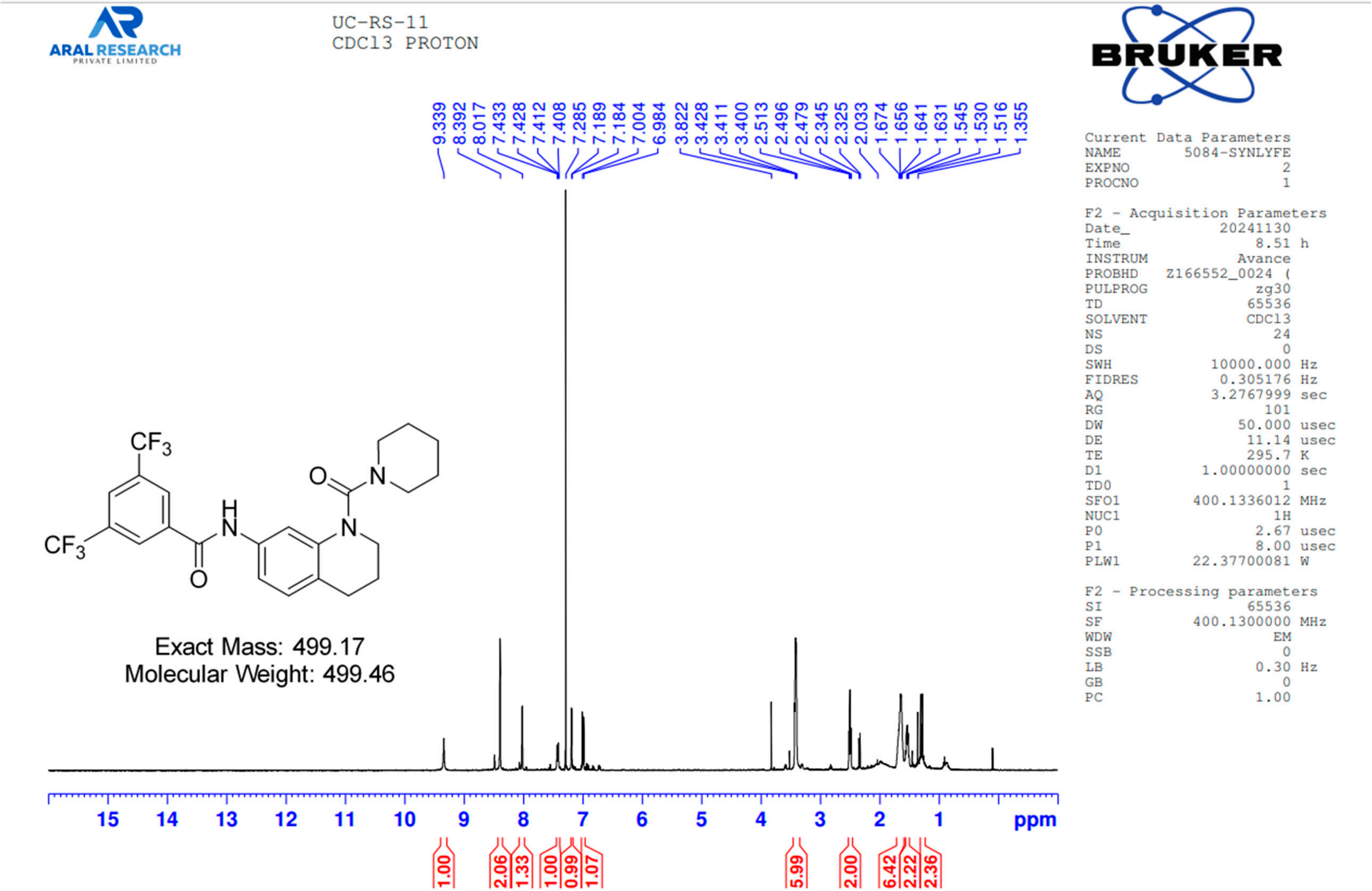

8.3. ^{13}C NMR of compound 10h

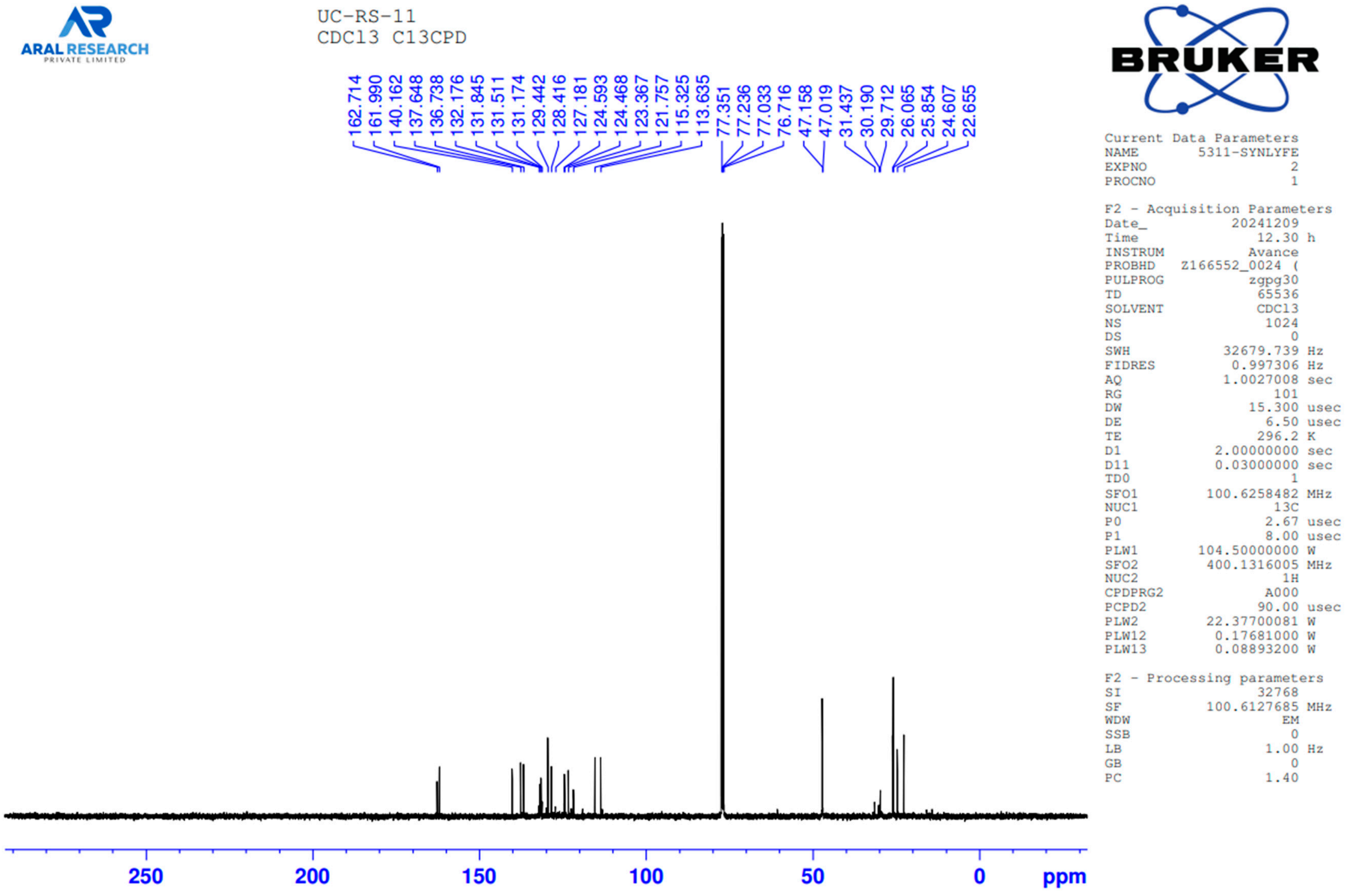

8.4. HPLC chromatogram of compound 10h

Sample Name: 10h

Acquired by	: Admin
Date Acquired	: 1/16/2024 5:34:55 PM
Sample ID	: O2Hr
Vial#	: 11
Injection Volume	: 10
Data File	: 10h_.lcd
Method file	:250_SUNFIRE_NORMAL METHOD.lcm

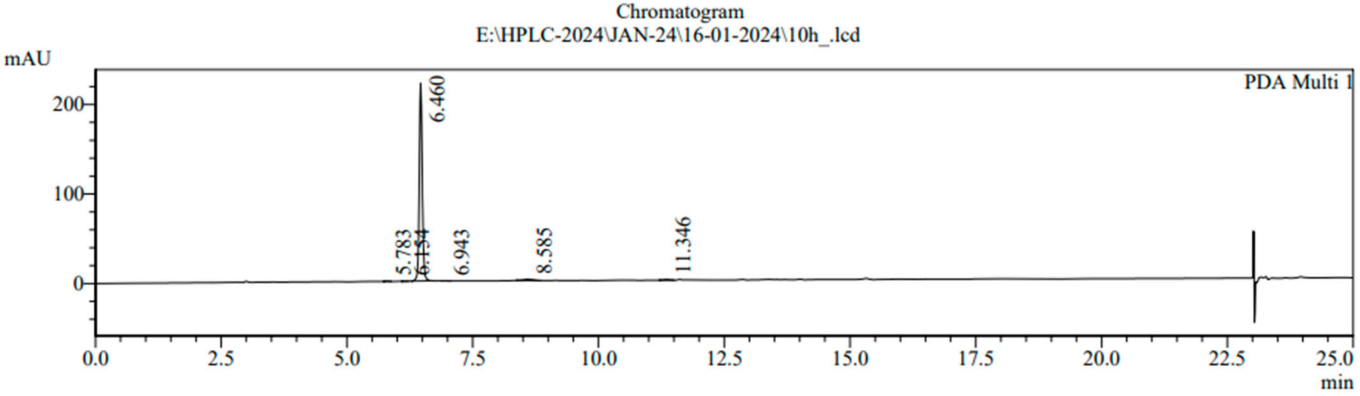

1 PDA Multi 1 / 307nm 4nm

PeakTable

PDA Ch1 307nm 4nm

Peak#	Ret. Time	Area	Height	Area %
1	5.783	1863	481	0.196
2	6.154	692	159	0.073
3	6.460	925837	221269	97.187
4	6.943	1577	385	0.166
5	8.585	17021	1377	1.787
6	11.346	5642	618	0.592
Total		952631	224287	100.000

Purity: 97%

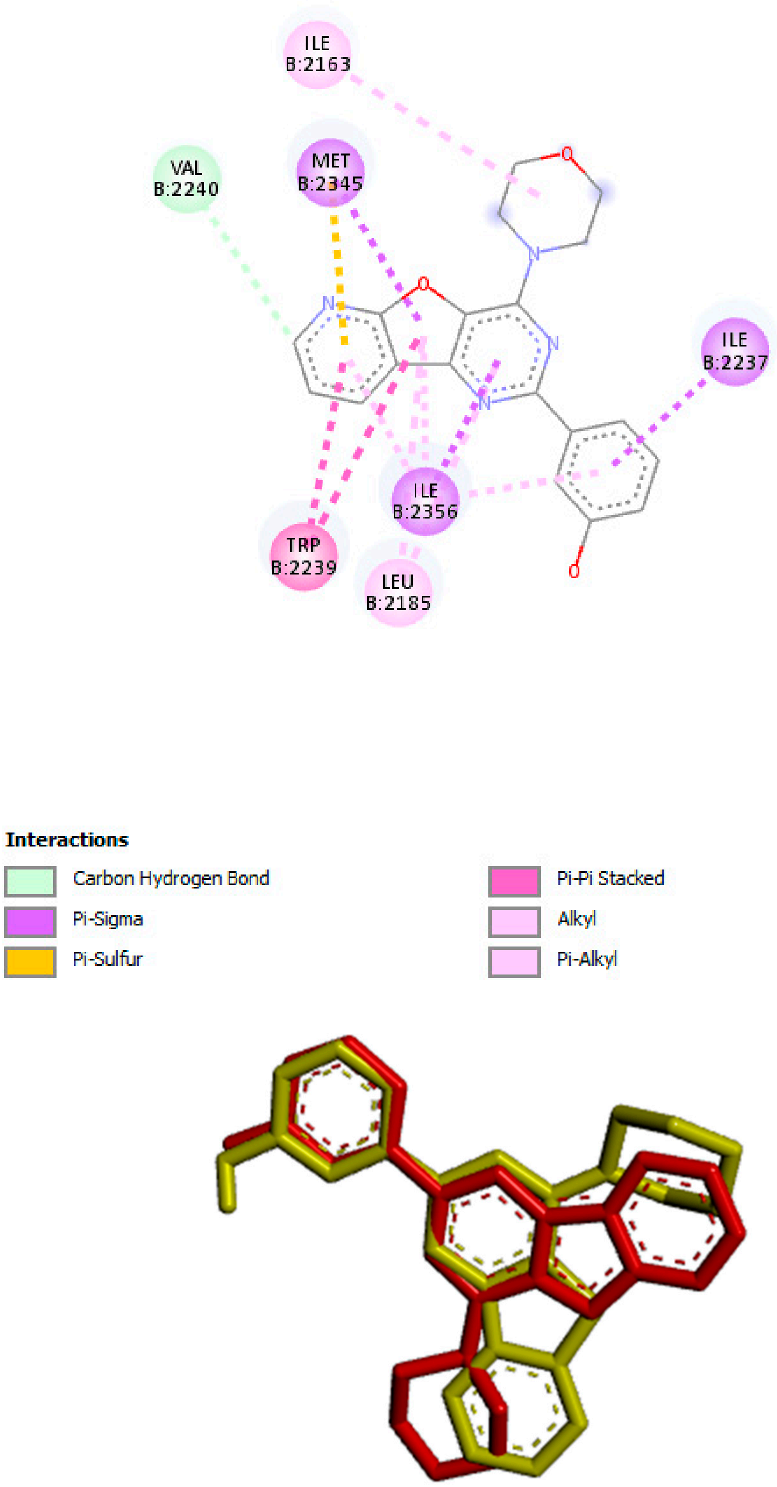

Figure S2: Ligand interaction diagram with the co-crystal ligand X6K (2D & 3D)
